# Exploring the research progression and evolutionary trends of gut microbiome and hypertension: a bibliometric analysis

**DOI:** 10.3389/fmicb.2025.1530857

**Published:** 2025-05-20

**Authors:** Xinyu Huang, Yingying Liu, Feng Jiang, Laiyun Xin, Zhenyuan Wang, Zhen Hua, Cong Wang, Lei Zhang, Jie Yu

**Affiliations:** ^1^The First Clinical Medical College of Shandong University of Traditional Chinese Medicine, Shandong, China; ^2^The Third Department of Cardiovascular Disease, Affiliated Hospital of Shandong University of Traditional Chinese Medicine, Shandong, China

**Keywords:** bibliometric analysis, research progression, gut microbiome, hypertension, CiteSpace, VOSviewer

## Abstract

Hypertension is one of the most common cardiovascular diseases, with multiple underlying mechanisms. The gut microbiome, as one of the most important microbial communities in the human body, plays a significant role in the development of various diseases, including hypertension. While numerous studies have explored the relationship between gut microbiome and hypertension from different perspectives, there has been no bibliometric study in this field. Therefore, a bibliometric analysis is crucial for accurately assessing and summarizing the current research status. The analysis indicates that the number of publications in this field has steadily increased in recent years, with China and the United States leading in development. The journal Nutrients has the highest number of published papers, and Marques, Francine Z is a prominent figure with significant contributions and influence in this field. Co-occurrence and trend analysis suggest that the main research hotspots include the relationship between gut microbiome and hypertension, mechanisms by which gut microbiome promotes hypertension, and new therapeutic strategies targeting gut microbiome to improve hypertension. Future research trends may focus on expanding new metabolites or measurement techniques, building databases of human and animal gut microbiota and their metabolites, and developing new drugs targeting gut microbiota for hypertension. In summary, this study visually demonstrates the dynamic changes in research hotspots, revealing new patterns and focuses in the field, and aims to provide new insights for clinical work on hypertension.

## 1 Introduction

Hypertension, as a serious chronic disease, is also one of the major risk factors for global mortality and disability. Epidemiological data show that the global prevalence of hypertension reached 33% in 2019. With the changing age demographics, the proportion of adults and the elderly has increased, with the number of adults aged 30–79 with hypertension rising from 650 million in 1990 to 1.3 billion in 2019 (Kario et al., 2024). In 2019, hypertension was responsible for as many as 10.8 million deaths (GBD 2019 Risk Factors Collaborators, 2020). Research indicates that hypertension is more prevalent in low- and middle-income countries (GBD 2019 Risk Factors Collaborators, 2020). A collaborative study encompassing 61 prospective observational studies found that for every 20 mmHg increase in systolic blood pressure (SBP) and every 10 mmHg increase in diastolic blood pressure (DBP), the risk of ischemic heart disease and stroke doubles (Lewington et al., 2002). Similarly, an Asia-Pacific cohort study observed that for individuals aged 55–64, each 10 mmHg increase in SBP was associated with a 45% increased risk of ischemic heart disease and a 65% increased risk of ischemic or hemorrhagic stroke (Singh et al., 2013). However, due to the insidious nature of hypertension, awareness, control, and treatment rates remain low, with only half of the patients receiving treatment and only one-third achieving controlled blood pressure (Beaney et al., 2020). The complications of hypertension, such as ischemic heart disease, stroke, chronic kidney disease, and vascular kidney disease, place significant pressure on healthcare institutions and impose a heavy burden on families and society. However, hypertension is considered a preventable risk factor. Therefore, actively seeking the causes and treatment strategies for hypertension has become a significant issue in the field of cardiovascular and cerebrovascular research.

Hypertension is influenced by multiple factors, including genetics and environment, making it complex and heterogeneous (Oparil et al., 2018). Most researchers view its etiology as a mosaic of multiple causes (Page, 1949). Genetic genomics has identified a range of gene loci and pathways associated with hypertension, revealing the direct impact of genetic factors (Keaton et al., 2024). Environmental factors such as high salt intake, alcohol consumption, and lack of exercise are also significant contributors (Li B. et al., 2021). The annual increase in hypertension rates is closely related to changes in human dietary patterns, including low fiber, high salt, and high-fat diets, alcohol consumption, and constipation, which can directly affect the gastrointestinal tract and its microbiota. The gut microbiome is one of the most important microbial communities in the human body, comprising over 1,500 species from more than 50 phyla (Gilbert et al., 2018). The gut microbiome includes bacteria, fungi, viruses, and protozoa, and can be categorized into probiotics, pathogenic bacteria, and neutral bacteria based on their effects on the body (Integrative HMP (iHMP) Research Network Consortium, 2019). After millions of years of evolution, these microorganisms have developed interdependencies with each other and the host, participating in metabolism, energy conversion, and immune regulation (Integrative HMP (iHMP) Research Network Consortium, 2019). Under normal physiological conditions, gut microbiome maintains a relative balance of species and numbers. Dysbiosis of the gut microbiome can trigger various diseases (Chen et al., 2021). Increasing research suggests that gut microbiome and its metabolites play a crucial role in the development of hypertension and that modulating gut microbiome could be a new target for hypertension treatment (Nakai et al., 2021; Kim S. et al., 2018; Yang et al., 2015), offering new perspectives for managing the condition.

**Figure 1:**
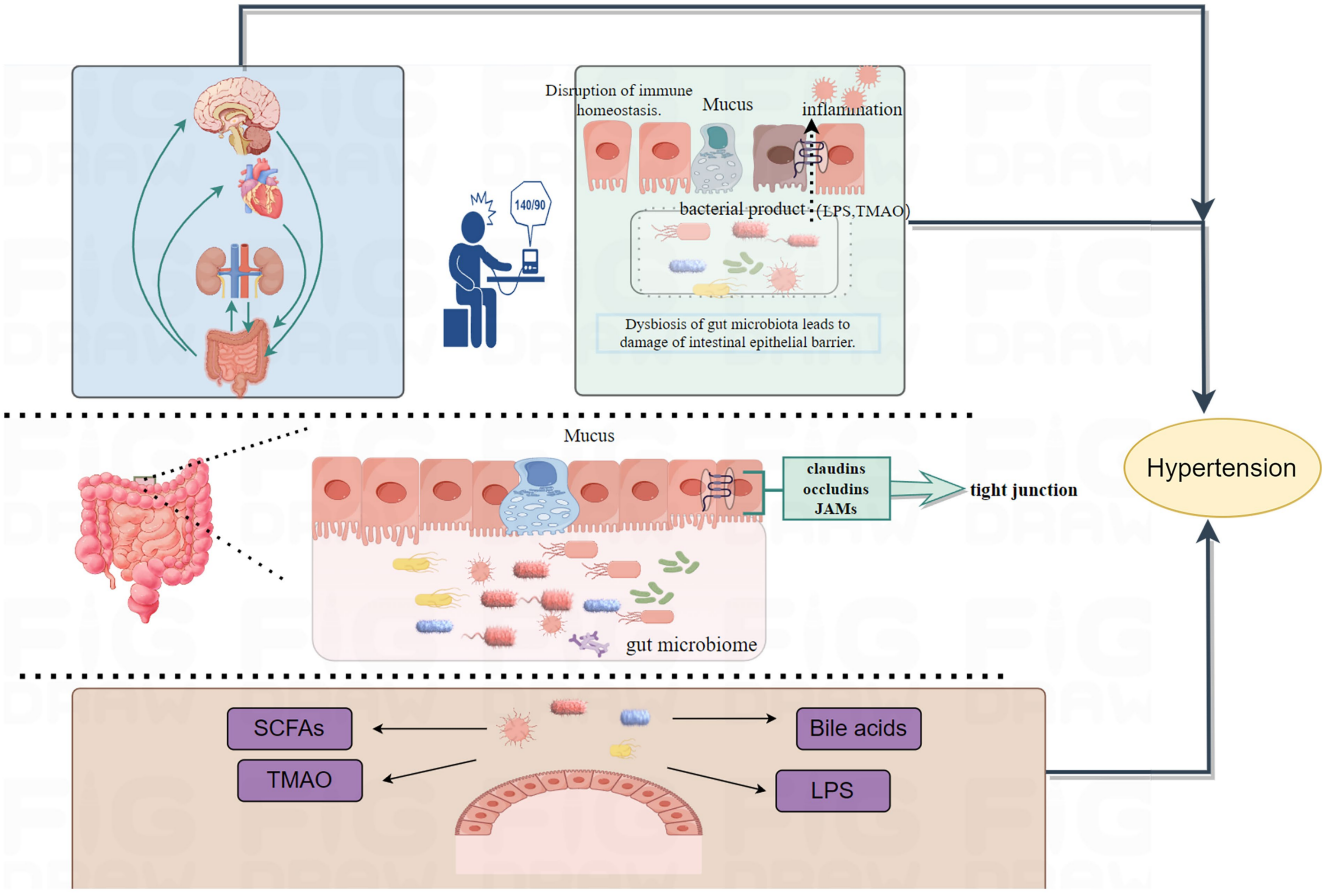
Mechanisms by which gut microbiota induce hypertension are illustrated. Gut microbiome affect blood pressure through multiple axes, including the gut-brain axis, gut-heart axis, and gut-kidney axis. Dysbiosis leads to damage of the intestinal barrier, activates inflammatory responses, and induces the onset of hypertension. Metabolites produced by gut microbiota can influence blood pressure either dependent on or independent of the host’s immune system.

The physiological impact of microorganisms on the host arises from the long-term evolutionary relationship between microorganisms and multicellular organisms. Evolution of multicellular organisms depends on ecosystems dominated by microorganisms, leading to a collaborative and interdependent existence between hosts and the communities formed by various microorganisms (Aburto and Cryan, 2024). Gut microbiome is primarily classified into phyla such as Firmicutes, Bacteroidetes, Actinobacteria, Proteobacteria, Verrucomicrobia, and Fusobacteria, with Firmicutes and Bacteroidetes being the dominant phyla, accounting for 80–90% of all bacteria (Zemin et al., 2018). Bacteroidetes mainly produce acetic and propionic acids, while Firmicutes mainly produce butyrate (Macfarlane and Macfarlane, 2003). As the largest microbial community in the human body, any changes in the gut microbiome can affect the host through various mechanisms and cascade effects. Studies have shown that an increase in Firmicutes and a decrease in Bacteroidetes are closely associated with hypertension, with the ratio of Firmicutes to Bacteroidetes in spontaneously hypertensive rats (SHR) being five times higher than in normal rats (Yang et al., 2015; Adnan et al., 2017). The relationship between gut microbiome and hypertension is gaining attention from researchers. Li et al. (2017) conducted longitudinal studies using metagenomics and metabolomics on the gut microbiome of hypertensive patients, finding that the microbiome of prehypertensive and hypertensive patients was highly similar, and differed from that of healthy individuals, showing relevant changes in gut microbiome associated with hypertension. The biological mechanisms between gut microbiome and hypertension are highly complex and cannot be explained by a single biological theory. However, researchers have preliminarily identified some general patterns (shown in Figure 1). Gut microbiome influences organs function through the gut-brain axis, gut-heart axis, and gut-kidney axis (Aburto and Cryan, 2024; Shi et al., 2024; Majumder et al., 2024), all of which are closely related to the development of hypertension. The gut is a key site for immune cell aggregation, and these immune cells play a crucial role in regulating hypertension (Richards et al., 2022). Additionally, metabolites produced by the gut microbiota are important mediators of communication between gut bacteria and the host, potentially affecting host blood pressure either dependently or independently of the host’s immune system (Poll et al., 2020). Dysbiosis can also damage the intestinal epithelial barrier, trigger inflammatory responses, and participate in blood pressure regulation through vascular morphology and function, as well as autonomic nervous system activity (O'Donnell et al., 2023). Advances in modern science and technology have significantly enhanced the dimensions and understanding of gut microbiome research. Current evidences highlight the close relationship between gut microbiome and hypertension, with some scholars suggesting that, in a certain sense, hypertension could be considered a bacterial disease (Oyama and Node, 2019). Therefore regulating gut microbiome has also become a new idea in the treatment of hypertension.

**Figure 2:**
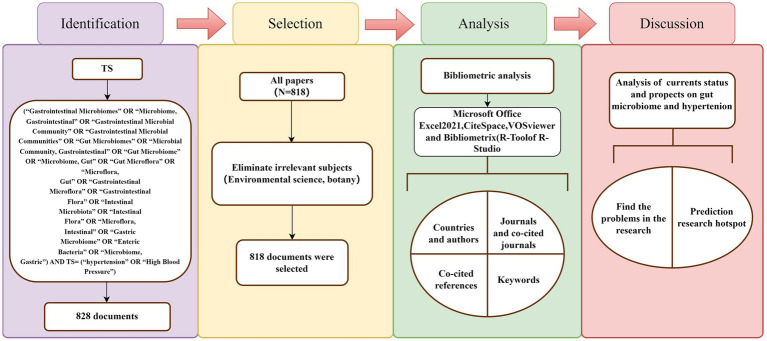
Flowchart of the literature-screening process and research framework.

Bibliometrics is a comprehensive approach that uses mathematical and statistical techniques to conduct both quantitative and qualitative analyses of literature (Ellegaard and Wallin, 2015). By dynamically analyzing specific information about authors, countries, institutions, journals, and references over time from various perspectives, bibliometrics provides insights into the research development, distribution patterns, hot topics, and frontiers within a particular field. It has become one of the popular techniques for evaluating the quality and impact of academic work in a given field (Ellegaard and Wallin, 2015; Hassan and Duarte, 2024). Although bibliometrics is widely applied across many academic disciplines, a search of literature related to gut microbiome and hypertension reveals that while there are many studies exploring different aspects of this field, there is a lack of bibliometric analysis of the research status. In this study, we used CiteSpace 6.2.R6, VOSviewer 1.6.18, and the “bibliometrix” package in R 4.3.2 to create scientific knowledge maps and analyze published literature from 1999 to 2024. This review summarizes the academic characteristics of the literature related to gut microbiome and hypertension and visually presents its dynamic evolution trends. We believe this study provides researchers in the field with a broader understanding and helps explore future research directions (Figure 2).

## 2 Materials and methods

### 2.1 Data collection and search strategy

**Figure 3:**
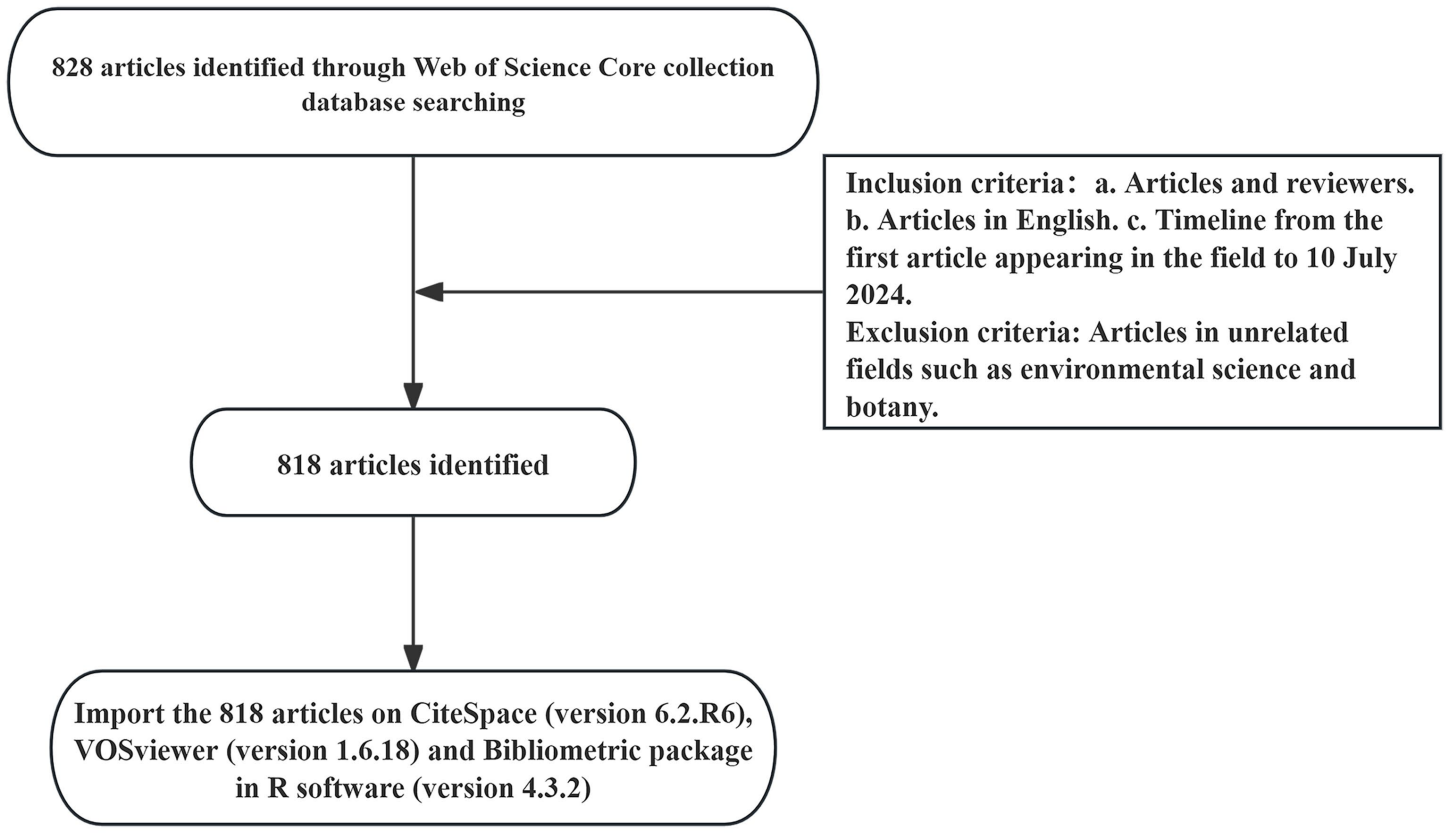
Flowchart of the literature search and screening procedures used in the study.

The Web of Science is the most authoritative global database for scientific citations, covering multidisciplinary information across the humanities, natural sciences, biomedicine, and social sciences. Due to its comprehensive bibliometric indicators, including publications and references, we selected Web of Science Core Collection as our data source, indexing SSCI and SCIE. The search strategy was TS = (“Gastrointestinal Microbiomes” OR “Microbiome, Gastrointestinal” OR “Gastrointestinal Microbial Community” OR “Gastrointestinal Microbial Communities” OR “Gut Microbiomes” OR “Microbial Community, Gastrointestinal” OR “Gut Microbiome” OR “Microbiome, Gut” OR “Gut Microflora” OR “Microflora, Gut” OR “Gastrointestinal Microflora” OR “Gastrointestinal Flora” OR “Intestinal Microbiota” OR “Intestinal Flora” OR “Microflora, Intestinal” OR “Gastric Microbiome” OR “Enteric Bacteria” OR “Microbiome, Gastric”) AND TS = (“hypertension” OR “High Blood Pressure”). To avoid biases from daily data updates, the time span was set from the publication date of the first relevant article to July 10, 2024. A total of 828 articles were obtained. This study excluded irrelevant disciplines such as environmental science and botany. Including literature from these fields could introduce a large amount of unrelated data, leading to a deviation in the co-occurrence network from the core topic and reducing the accuracy of the analysis. Therefore, a total of 818 articles were retained, as shown in the flowchart in Figure 3.

### 2.2 Data analysis and visualization

In this study, we utilized the analytical features of the Web of Science database to summarize the number of publications and the average citation count. We downloaded the literature records and citation reference data, naming the files as “download_1-500.txt” and “download_501-818.txt.” These files were then imported into CiteSpace 6.2.R6, VOSviewer 1.6.18, and the “bibliometrix” package in R 4.3.2 for data analysis and knowledge map generation. CiteSpace, created by Chaomei Chen from Drexel University, is a software for visualizing scientific publication trends and patterns. It uses set theory for data standardization and similarity measurement, and helps researchers understand the evolution of knowledge over time through timeline charts, providing insights into the field’s development (Chen, 2006). The CiteSpace parameters are set as follows: time slice (2000–2023), year per slice (2), term source (entire selection), node type (e.g., author, institution, and keyword), and selection criteria (top *N* = 50). Other parameters are kept at their default settings, and after manually excluding irrelevant terms or merging synonyms, the network map is visualized. VOSviewer, developed by Ludo Waltman and Nees Jan van Eck from Leiden University, is a free tool based on visualization technology for processing bibliometric data. It uses probabilistic data standardization methods and offers three types of visual maps: network view, overlay view, and density view (Eck and Waltman, 2010). The VOSviewer settings are as follows: the analysis objects are selected as keyword, author, or institution, etc. Irrelevant terms are manually excluded or synonyms are merged, and the Minimum number of occurrences is set to generate the co-occurrence network. The R package “bibliometric” provides a suite of tools for bibliometric research, built on the open-source R statistical programming environment. It includes comprehensive functions for bibliometric analysis and scientific evaluation, such as data import, data cleaning, indicator calculation, and visualization (Aria and Cuccurullo, 2018). The “bibliometrix” online analysis platform is primarily used to analyze the country distribution of literature and inter-country collaboration relationships. The data is exported and then used with “OriginPro 2024 10.1.0.178” software to create a country collaboration chord diagram. By leveraging the complementary strengths of these three tools, we visually present the basic output, research trends, and new hotspots in the fields of gut microbiome and hypertension research. Overall, these analytical tools provide impartial and diverse perspectives on the literature in this area.

### 2.3 Ethical considerations

All data in this study were obtained from the Web of Science Core Collection and did not involve any patients or public contributions.

## 3 Results

### 3.1 Annual publication and citation trends

**Figure 4:**
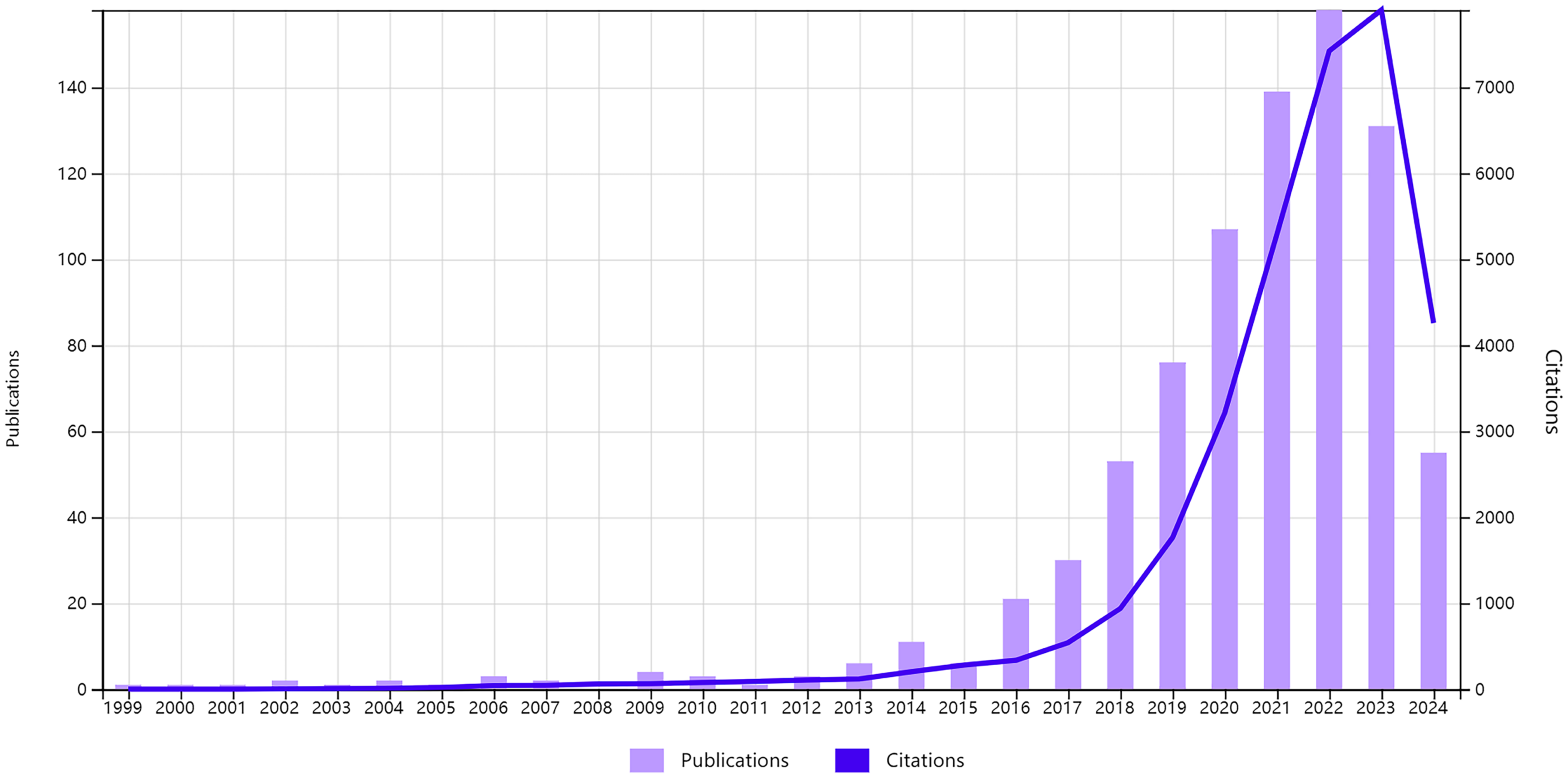
The combination chart presents the annual publication count and average citation per year of gut microbiome and hypertension from 1999 to 2024.

From 1999 to 2024, a total of 818 researches and review articles discussing the relationship between gut microbiome and hypertension were published. The bar chart in Figure 4 showed the number of articles each year from 1999 to 2024. Since the early 21st century, the number of publications in this field has generally increased. However, we observed that only 47 articles were published between 1999 and 2015. With growing interest from scholars, there has been a significant rise in the number of publications since 2016. The year with the highest number of articles published was 2022, with a total of 158 articles (data as of July 10, 2024). The line chart shows the average annual citation frequency of related articles from 1999 to 2024. From 1999 to 2013, the average annual citation frequency remained relatively low. From 2014 to 2017, it exhibited a slow upward trend. Since 2018, there has been a significant increase, with citations peaking at 7,899 in 2023.

### 3.2 Distribution of countries/regions and institutions

**Figure 5:**
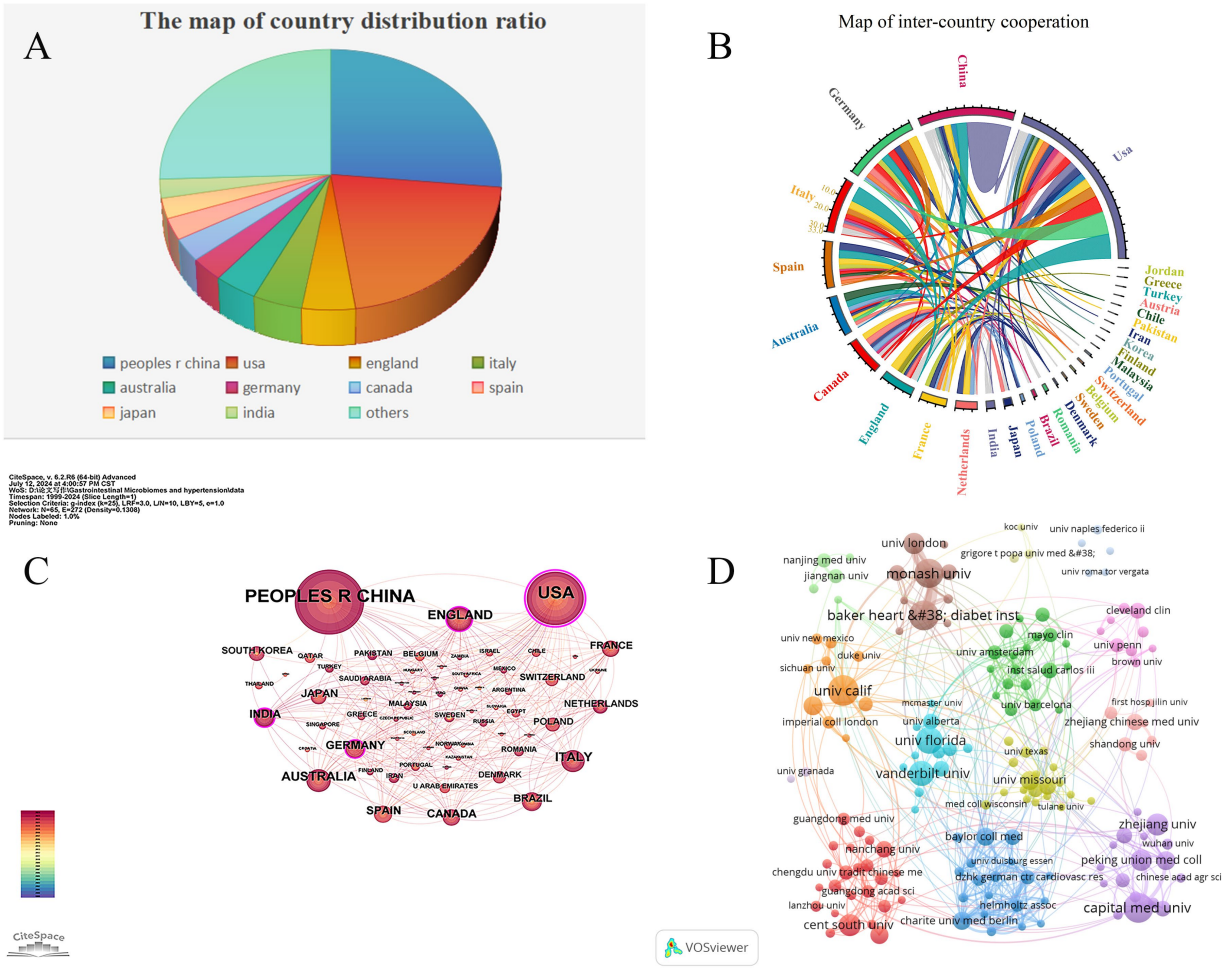
Cooperation map of countries/regions and institutions in gut microbiome and Hypertension. **(A)** The pie chart displays the proportional distribution of publication outputs in the gut microbiome and hypertension research field across countries/regions, with distinct color codes representing specific nations. Larger color segments indicate higher publication volumes from the corresponding country/region. **(B)** The chord diagram illustrates the cooperative relationships between countries/regions in the field of gut microbiome and hypertension research field. The lines’ width represents the connections’ strength, with wider bands indicating stronger collaborations. Countries/regions with wider bands and more diverse colors on the lines indicate that they have established collaborations with more countries/regions. **(C)** The collaborative network of countries/regions was visualized using CiteSpace. **(D)** The collaborative network of institutions was visualized using VOSviewer. This map visualizes the cooperative relationships and correlations of research between institutions.

Table 1 lists the top 10 countries/regions with the highest number of published articles, along with their citation frequencies and total connection strengths. Figure 5A shows the proportion of articles published by these top 10 countries. Figure 5C uses Citespace software to present a visual map of publication trends across different countries. China is the leading country in terms of the number of published articles (295), followed by the United States (235), the United Kingdom (54), and Italy (50), which account for 36.06, 28.73, 6.6, and 6.11% of the total publication volume, respectively, surpassing 60% of the total. This indicates that these countries play a significant role in the field and highlights the uneven research development across different countries. Notably, the United States has accumulated 13,807 citations, more than double China’s 6,986, suggesting that the U.S. leads in both the quantity and quality of published articles. Furthermore, Germany and Spain have high average citation rates of 105.21 and 95.93, respectively, and also rank among the top 10 in publication volume, indicating their significant impact in the field. Similarly, in institutional analysis, the top three institutions in terms of publication volume are the University of California system in the U.S. (47), Capital Medical University in China (38), and the Baker Heart and Diabetes Institute in Australia (37). All these institutions have accumulated over 1,200 citations (Table 2), indicating their prominent positions in the fields of gut microbiome and hypertension.

**Table 1:** Top 10 most productive countries/regions in gut microbiome and hypertension.

Rank	Countries/regions	Record count	Percentage (%)	Average per item	Citations	Total link strength	Centrality
1	PEOPLES R CHINA	295	36.06	23.68	6,986	65	0.09
2	UNITED STATES	235	28.73	58.75	13,807	163	0.68
3	ENGLAND	54	6.60	58.43	3,155	76	0.2
4	ITALY	50	6.11	58.74	2,937	47	0.06
5	AUSTRALIA	43	5.26	61.93	2,663	38	0.1
6	GERMANY	33	4.03	105.21	3,472	72	0.17
7	CANADA	32	3.91	52.13	1,668	49	0.03
8	SPAIN	30	3.67	95.93	2,878	47	0.04
9	JAPAN	28	3.42	58.57	1,640	16	0.02
10	INDIA	26	3.18	20.65	537	29	0.05

**Table 2:** Top 10 institutions based on publications.

Rank	Organization	Documents	Citations	Total link strength	Centrality
1	University of California System	47	1,836	37	0.19
2	Capital Medical University	38	1,273	28	0.11
3	Baker Heart and Diabetes Institute	37	1,259	49	0.04
4	Monash University	32	1,257	48	0.02
5	University of Florida	28	983	12	0.03
6	Vanderbilt University	22	1,393	16	0.04
7	Central South University	20	335	10	0.01
8	Zhejiang University	17	1,222	13	0.01
9	University of London	15	1,649	36	0.07
10	Peking Union Medical College	13	1,386	28	0.05

Figure 5B illustrates the collaboration network of the top 30 countries in the fields of gut microbiome and hypertension. The United States leads in terms of collaboration, working with 26 countries including China, the United Kingdom, Germany, and Italy. China follows, collaborating with 15 countries such as the U.S., the U.K., and South Korea. Notably, the collaboration between China and the U.S. is the closest, with 29 joint publications. Such international collaboration fosters the growth of the field and facilitates the exchange and advancement of scientific knowledge among nations. Using VOSviewer and Pjake software, the institutional clustering was visualized, as shown in Figure 5D, which displays the global distribution of gut microbiome and hypertension research and identifies potential collaboration opportunities between institutions. Collaborations are grouped into 13 clusters, reflecting the cooperation levels and connections in gut microbiome and hypertension research. Nodes within each cluster are more tightly connected, while connections between clusters are sparser. The University of California system, as the institution with the highest publication volume, also has the most collaborations, partnering with institutions such as New York University, Harvard University, University College London, and Peking University. In China, Capital Medical University maintains close collaborations with Peking Union Medical College, China Medical University, and Zhejiang University. Some institutions, like the University of Granada and the University of Naples Federico II, have relatively isolated connections with other institutions.

### 3.3 Analysis of authors and co-cited authors

Analyzing the authors of literature provides insight into the leading scholars and core research forces in a given field. According to Price’s Law, the minimum number of publications for a core author in a field is given by m = 0.749 × √nmax () (where nmax is the maximum number of publications by any author) ≈ 3.43. Therefore, authors with three or more publications can be considered core authors in that field. There are a total of 124 core authors, indicating that the field of gut microbiome and hypertension research has established a stable research base.

Table 3 lists the top 10 authors by publication volume. The most prolific author is Francine Z. Marques from Baker Heart and Diabetes Institute, with 21 publications and a total of 1,257 citations. She is followed by Jing Li from Capital Medical University, with 14 publications and 1,170 citations, and David M. Kaye from Monash University, with 12 publications and 1,031 citations. Notably, Marques, Francine Z, who has the highest number of publications, also has the highest citation count, highlighting her significant impact in the field.

**Table 3:** Top 10 authors in gut microbiome and hypertension.

Rank	Author	Record count	% of 818	Citations	Average per item	H-index	Affiliations	Total link strength
1	Marques, Francine Z	21	2.57	1,257	59.86	13	Baker Heart and Diabetes Institute	82
2	Li, Jing	14	1.71	1,170	83.57	10	Capital Medical University	57
3	Kaye, David M	12	1.47	1,031	85.92	9	Monash University	66
4	Pepine, Carl J	11	1.34	768	69.82	8	University of Florida	47
5	Raizada, Mohan K	11	1.34	768	69.82	8	University of Florida	47
6	Tain, You-Lin	11	1.34	217	69.82	10	Chang Gung University	30
7	Richards, Elaine M	10	1.22	679	67.9	8	University of Florida	45
8	Hsu, Chien-Ning	9	1.10	177	19.67	8	Kaohsiung Medical University	28
9	Kirabo, Annet	9	1.10	305	33.89	7	Vanderbilt University	20
10	Cai, Jun	8	0.98	1,156	144.5	7	China Academy of Chinese Medical Sciences	26

**Figure 6:**
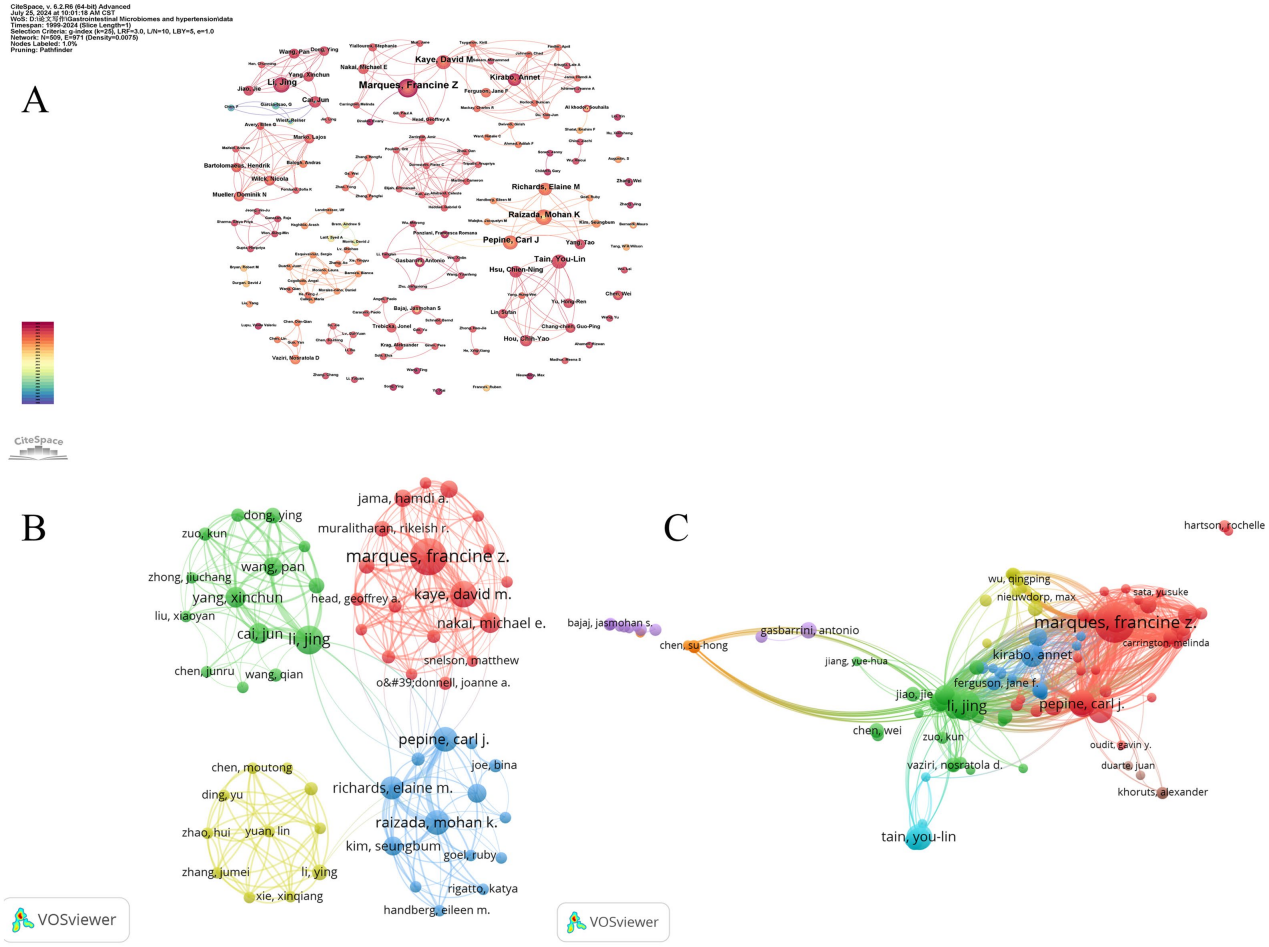
The collaborative network of co-authors and co-cited authors. **(A)** The collaborative network of co-authors was visualized using CiteSpce. Each node represents an author, with the size of the node indicating the number of publications by the author. The connections between the nodes represent collaborative relationships, with thicker lines indicating closer collaboration. **(B)** The collaborative network of co-authors was visualized using VOSviewer. Each node, represented by a different color, corresponds to an author belonging to a specific cluster. The size of each node indicates the frequency of co-occurrence, while the connections between nodes represent the co-occurrence relationships among authors. **(C)** The collaborative network of co-cited authors was visualized using VOSviewer. This map visualizes the co-cited relationships between authors, highlighting the similarities in their research interests and contributions.

The author collaboration network is visualized using both Citespace (Figure 6A) and VOSviewer (Figure 6B). In Figure 6A, the size of the nodes represents the number of publications by the authors, and different colors indicate different years, ranging from purple in 1999 to red in 2024. Based on the analysis of collaborating authors, VOSviewer categorizes them into four groups, with each color representing a distinct group of authors. Each node in the figure represents an individual author, with the size of the circle indicating the number of publications. The lines between nodes denote collaboration relationships, with thicker lines indicating stronger collaborations. As shown, Francine Z. Marques plays a crucial role in the collaboration network and is a key contributor to the field. It is also notable that fixed and close collaborations have formed among the authors, with clear cooperation between Marques and David M. Kaye. In contrast, connections between different clusters are sparse and less significant.

Co-citation author analysis involves examining how often a third author cites two other authors. A higher co-citation frequency indicates similar research focuses and interests, as well as higher article quality. By studying authors with many publications and high co-citation frequencies in gut microbiome and hypertension research, we can gain insights into the research expertise of scholars in this field and identify key areas of research in gut microbiome and hypertension. Figure 6C displays eight clusters, with interconnections between them suggesting related research among scholars. The four main clusters are: (1) Authors like Francine Z. Marques and Carl J. Pepine (red cluster) focusing on mechanisms of gut microbiome -induced hypertension and the role of dietary fibers and short-chain fatty acids; (2) Li Jing and other scholars (green cluster) concentrating on the gut-brain axis in hypertension development; (3) Annet Kirabo and other scholars (blue cluster) investigating the inflammatory immune mechanisms of gut microbiome-induced hypertension; and (4) You-Lin Tain and other scholars (light blue cluster) studying the intrinsic relationship between dysbiosis of gut microbiome and hypertension of developmental origins.

### 3.4 The distribution of journals

**Figure 7:**
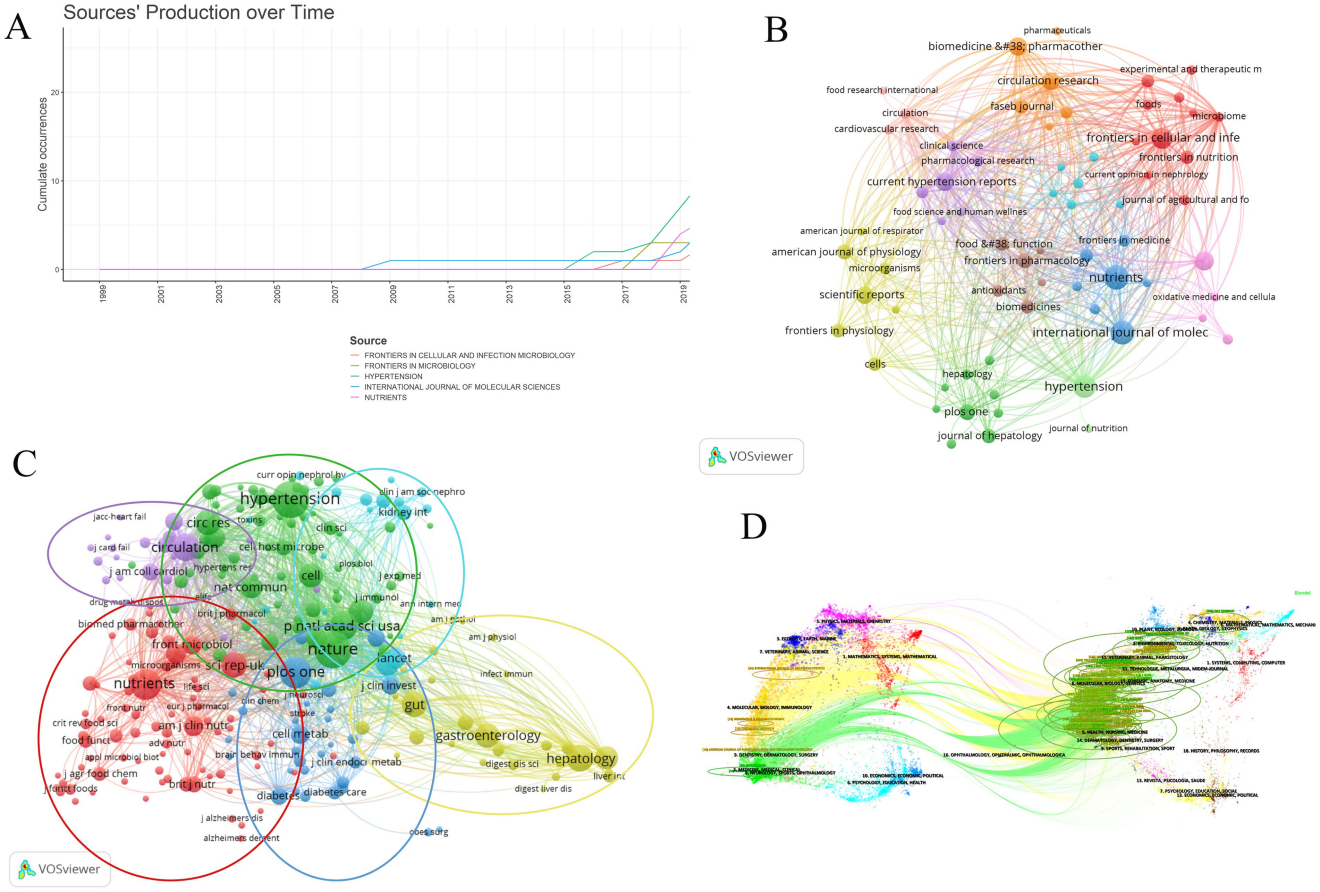
The analysis of academic journals related to gut microbiome and hypertension. **(A)** Trends in the number of articles published in the top five journals over time. **(B)** The visualization of journal collaborations in VOSviewer analyzes the collaborative relationships between journals. The size of the nodes corresponds to the frequency of these journals’ presence in the network. **(C)** The visualization of journal citations in VOSviewer allows for exploring the connections between journals. The size of the nodes signifies the frequency of their citations, indicating the importance and influence of the journals within the network. **(D)** The dual-map overlay of journals illustrates the citation relationships between citing journals on the left and cited journals on the right. The width of the connecting lines signifies the strength of the citation relationships.

Table 4 shows the top ten journals with the highest number of publications, representing active and influential journals in gut microbiome and hypertension research. The journal with the most publications is NUTRIENTS (26), followed by INTERNATIONAL JOURNAL OF MOLECULAR SCIENCES (24), and HYPERTENSION (23). Figure 7A shows the publication volume trend over time for the top five journals ranked by publication volume. Of the top ten journals in this field, 9 are classified as Q1 in JCR 2023, indicating their high impact. Prolific journals often reflect the research status and hot topics in the field, but highly cited journals, as knowledge repositories, point to broader and limitless potential directions. Table 5 presents the top 10 journals by citation frequency. Notably, all 10 journals are Q1 in JCR 2023, with impact factors exceeding 10, including Nature (50.5), Circulation (35.5), Gut (23.0), Circulation Research (16.5), and Cell (45.5). These are outstanding multidisciplinary journals that provide high-level references and significantly contribute to the development of the field.

**Table 4:** The top 10 journals in gut microbiome and hypertension.

Rank	Sources	*N* (%)	IF^a^ (2023)	JCR^b^ (2023)
1	NUTRIENTS	26 (3.19)	4.800	Q1
2	INTERATIONAL JOURNAL OF MOLECULAR SCIENCES	24 (2.93)	4.900	Q1
3	HYPERTENSION	23 (2.81)	6.900	Q1
4	FRONTIERS IN CELLULAR AND INFECTION MICCROBIOLOGY	18 (2.20)	4.600	Q1
5	FRONTIERS IN MICROBIOLOGY	15 (1.83)	4.000	Q2
6	BIOMEDICNE & PHARMACOTHERAPY	14 (1.71)	6.900	Q1
7	CURRENT HYPERTENSION REPORTS	14 (1.71)	3.900	Q1
8	CIRCULATION RESEARCH	13 (1.59)	16.50	Q1
9	SCIENTIFIC REPORTS	13 (1.59)	3.800	Q1
10	PLOS ONE	11 (1.34)	2.900	Q1

a IF, impact factor.

b JCR, journal citation reports.

**Table 5:** The top 10 co-citation journals in gut microbiome and hypertension.

Rank	Sources	*N*	IF^a^ (2023)	JCR^b^ (2023)
1	NATURE	1,798	50.50	Q1
2	HYPERTENSION	1,756	6.900	Q1
3	PLOS ONE	1,310	2.900	Q1
4	NUTRIENTS	1,217	4.800	Q1
5	CIRCULATION	1,001	35.50	Q1
6	PROCEEDINGS OF THE NATIONAL ACADEMY OF SCIENCES OF THE UNITEDS STATES OF AMERICA	989	9.400	Q1
7	HEPATOLOGY	977	3.900	Q1
8	GUT	875	23.00	Q1
9	CIRCULATION RESEARCH	862	16.500	Q1
10	CELL	748	45.500	Q1

a IF, impact factor.

b JCR, journal citation reports.

The visualization map generated by VOSviewer illustrates the journals publishing gut microbiome and hypertension-related literature and their interrelationships (Figure 7B). The clustering of journals is based on their similarities, resulting in 11 clusters. The red cluster mainly consists of journals focusing on microbiome and nutrition research (e.g., Frontiers in Cellular and Infection, Microbiology, Microbiome, Frontiers in Nutrition, Foods). The orange cluster includes biomedical journals focusing on disease mechanisms and pharmacology (e.g., Biomedicine and Pharmacotherapy, Circulation Research, FASEB Journal). The yellow cluster comprises journals in the life sciences with broad research scope (e.g., Frontiers in Physiology, Microorganisms, Scientific Reports, Cells). The dark green cluster consists of journals related to liver research (e.g., Hepatology, Journal of Hepatology, PLOS ONE). The purple cluster includes journals focusing on recent advancements and pharmacology in hypertension research (e.g., Clinical Science, Current Hypertension Reports, Pharmacological Research). The orange cluster contains journals concentrating on drug research and the cardiovascular system (e.g., Biomedicine and Pharmacotherapy, FASEB Journal, Circulation Research, Pharmaceuticals).

Based on the frequency of common citations among journals, these journals are grouped into six clusters (Figure 7C), indicating similarities in their research focuses. The red cluster includes journals with a focus on nutritional science, including the impact of nutrition on health, nutritional assessment, and food chemistry (e.g., Nutrients, American Journal of Clinical Nutrition, British Journal of Nutrition, Journal of Agricultural and Food Chemistry, Food and Function). The blue cluster comprises journals that focus on disease mechanisms, metabolism, and immunology within the biomedical field, both basic and clinical research (e.g., PLOS ONE, The Lancet, Journal of Clinical Investigation, Cell Metabolism, Brain, Behavior, and Immunity). The yellow cluster is centered around research on the digestive system (e.g., Gut, Gastroenterology, Digestive Diseases and Sciences, Hepatology). The purple cluster consists of journals focusing on cardiovascular diseases (e.g., Circulation, Journal of Cardiac Failure, JACC: Heart Failure, Journal of the American College of Cardiology). The green cluster includes journals that address multidisciplinary fields, cardiovascular diseases like hypertension, as well as immunology and microbiology (e.g., Nature, Cell, Hypertension, Cell Host and Microbe, Journal of Immunology). The light blue cluster is made up of journals that focus on kidney health and related diseases (e.g., Kidney International, Clinical Journal of the American Society of Nephrology, Current Opinion in Nephrology and Hypertension, Annals of Internal Medicine).

Figure 7D illustrates a dual-map overlay of journals in the field, visually representing the distribution of citing and cited journals, the evolution of citation patterns, and shifts in research focus within these journals. This map highlights changes in academic publications and their citation patterns, helping readers clearly understand the relationships and dynamics among disciplines in gut microbiome and hypertension research. Journals on the left, which are citing sources, represent current research hotspots, while journals on the right, which are cited sources, reflect the knowledge base of the field. The colored bands connecting the two sides indicate the knowledge foundation and background of these research hotspots. Citing journals are primarily from molecular biology, immunology, medicine, clinical medicine, and other cutting-edge research areas. Cited journals mainly come from molecular biology, health, nursing, pharmacology, forensic anatomy, and nutrition disciplines.

### 3.5 Analysis of co-cited and cited references

**Figure 8:**
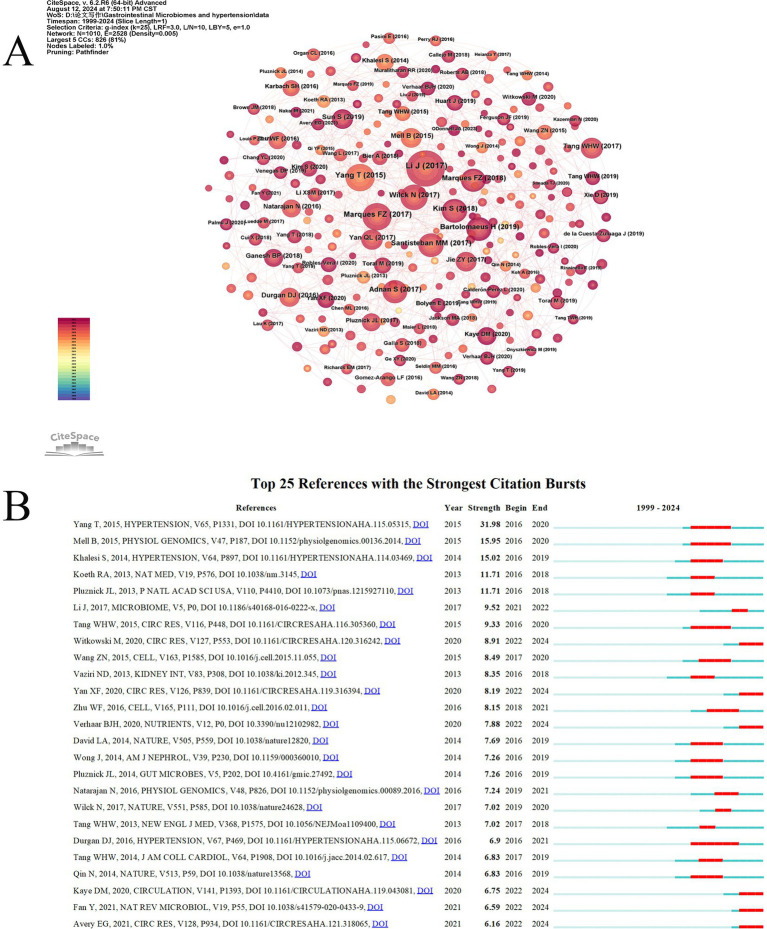
The visual analysis of highly cited references related to gut microbiome and hypertension. **(A)** The high-citation reference network diagram is generated by CiteSpace, with each node representing a cited reference. The larger the node, the higher the citation frequency of the reference. **(B)** The figure showcases the top 25 references with the most robust citation bursts, highlighting abrupt shifts in information represented by red outbreaks along the timeline. These bursts indicate rapid increases in citations and the emergence of essential questions or solutions in the field.

Co-citation occurs when two references are cited together by another paper. Table 6 shows the top 10 most frequently cited references, with the most cited being “Gut microbiota dysbiosis contributes to the development of hypertension,” authored by Jing Li and published in Microbiome (159 citations). The second most cited are “High Fiber Diet and Acetate Supplementation Change the Gut Microbiota and Prevent the Development of Hypertension and Heart Failure in DOCA-Salt Hypertensive Mice,” by Marques, Francine Z, published in Circulation (101 citations), and “Gut Dysbiosis Is Linked to Hypertension,” by Tao Yang, published in Hypertension (101 citations). The third is “Salt-responsive gut commensal modulates TH17 axis and disease,” by Nicola Wilck, published in Nature (82 citations). High co-citation counts indicate the significant impact of these papers in the field. Among the top 10 co-cited references, eight are experimental articles and two are review articles. Figure 8A shows the co-citation reference map. Based on the co-citation map, we further conducted a co-citation reference burst analysis. Citation burst refers to a significant increase in the citation frequency of a particular paper within a specific time period, surpassing the historical baseline level. Papers with high citation bursts may represent key research that introduces new theories, technological breakthroughs, or resolves controversies. Figure 8B presents the top 25 most prominent co-cited references. The reference with the highest prominence is “Gut Dysbiosis Is Linked to Hypertension,” published in Hypertension, with a prominence score of 31.98, indicating substantial scholarly attention from 2016 to 2020.

**Table 6:** Top 10 cited references of publications in gut microbiome and hypertension.

Rank	Author	Title	Journal	Co-citation	Centrality
1	Jing Li	Gut microbiota dysbiosis contributes to the development of hypertension	Microbiome	159	0
2	Marques, Francine Z	High Fiber Diet and Acetate Supplementation Change the Gut Microbiota and Prevent the Development of Hypertension and Heart Failure in DOCA-Salt Hypertensive Mice	Circulation	101	0.04
3	Tao Yang	Gut Dysbiosis Is Linked to Hypertension	Hypertension	101	0
4	Nicola Wilck	Salt-responsive gut commensal modulates TH17 axis and disease	Nature	82	0
5	Seungbum Kim	Imbalance of gut microbiome and intestinal epithelial barrier dysfunction in patients with high blood pressure	Clinical Science	77	0.02
6	Monica M. Santisteban	Hypertension-Linked Pathophysiological Alterations in the Gut	Circulation research	76	0
7	Hendrik Bartolomaeus	The Short-Chain Fatty Acid Propionate Protects from Hypertensive Cardiovascular Damage	Circulation	73	0.02
8	Marques, Francine Z	Beyond gut feelings: how the gut microbiota regulates blood pressure	Nature Reviews | Cardiology	69	0
9	Sareema Adnan	Alterations in the gut microbiota can elicit hypertension in rats	Physiolgenomics	69	0.01
10	W.H. Wilson Tang	Gut Microbiota in Cardiovascular Health and Disease	Circulation Research	56	0.01

### 3.6 A review of important academic achievements in the field of gut microbiome and hypertension

**Figure 9:**
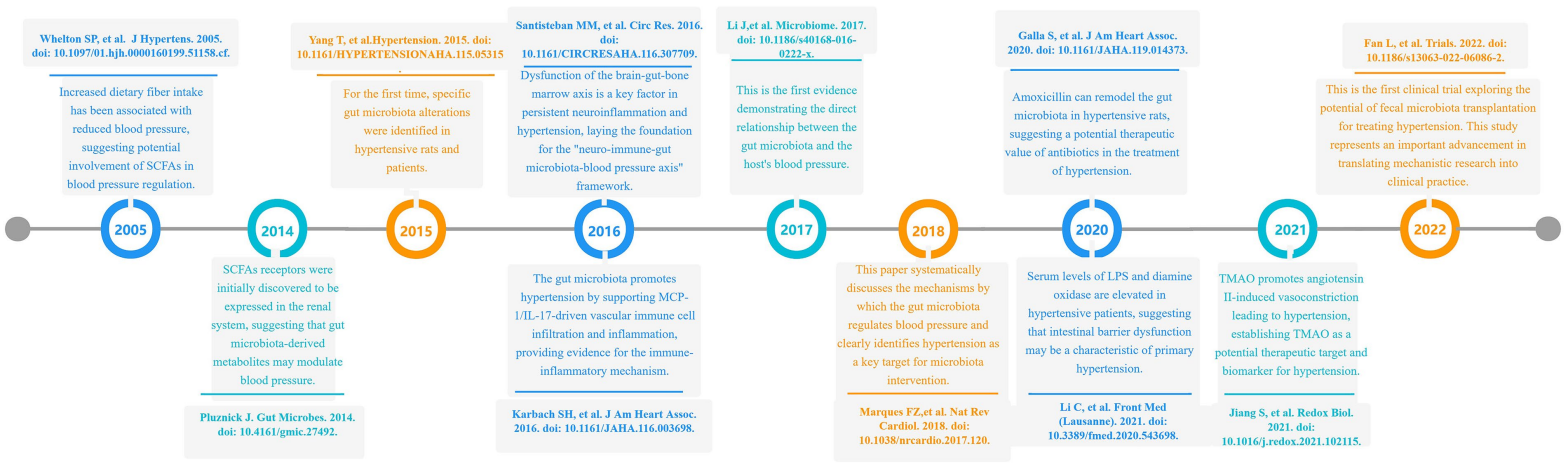
A chronological review of significant scientific achievements in the field of gut microbiota and hypertension research.

A 2005 study demonstrated that increased dietary fiber intake reduced blood pressure in hypertensive patients, suggesting the potential involvement of SCFAs in blood pressure regulation (Whelton et al., 2005). In 2014, Pluznick et al. first identified the renal expression of SCFAs receptors, implying that gut microbiota-derived metabolites may modulate blood pressure (Pluznick, 2014). In 2015, Tao Yang’s research directly observed specific microbial alterations in hypertensive rats and patients, characterized by an elevated Firmicutes/Bacteroidetes ratio and reduced abundance of acetate- and butyrate-producing bacteria (Yang et al., 2015). A 2016 investigation by Karbach et al. revealed that gut microbiota promotes vascular dysfunction and hypertension by supporting MCP-1/IL-17-driven vascular immune cell infiltration and inflammation, providing experimental evidence for immune-inflammatory mechanisms linking gut microbiota to hypertension (Karbach et al., 2016). The same year, Monica et al. systematically discussed the functional interplay among the brain, gut, and brain-gut-marrow axis in a review, proposing that their dysregulated interactions may drive sustained neuroinflammation and contribute to hypertension pathogenesis, thereby establishing the “neuro-immune-gut microbiota-blood pressure axis” framework (Santisteban et al., 2016). In 2017, Li et al. demonstrated through fecal microbiota transplantation (FMT) experiments that gut microbiota from hypertensive patients elevates blood pressure in germ-free mice, providing first direct evidence of microbiota-host blood pressure causality (Li et al., 2017). Marques et al. (2018) systematically reviewed gut microbiota-mediated blood pressure regulation mechanisms, designating hypertension as a priority target for microbiota-based interventions. A 2020 study of 106 Chinese hypertensive patients reported elevated serum lipopolysaccharide (LPS) and diamine oxidase (DAO, a biomarker of intestinal barrier function), suggesting intestinal barrier impairment as a potential feature of essential hypertension (Li et al., 2020). Galla et al. (2020) showed that amoxicillin remodels gut microbiota in hypertensive rats and exerts long-term antihypertensive effects, highlighting antibiotics’ therapeutic potential. Jiang et al. (2021) discovered that microbiota-derived trimethylamine N-oxide (TMAO) activates the PERK/EIF2α/CHOP/ERO1-α signaling pathway, inducing excessive ROS production and promoting angiotensin II-induced vasoconstriction to exacerbate hypertension, identifying TMAO as a novel therapeutic target and biomarker. Fan et al. (2022) conducted the first clinical trial exploring FMT for hypertension management, marking a critical transition from mechanistic research to clinical translation. The timeline is shown in Figure 9.

### 3.7 Keyword analysis

Keywords are essential for reflecting the themes and content of research papers. The frequency of keyword occurrence can indicate research priorities in a specific field and track changes in trending topics over time. Identifying frequently co-occurring keywords and visualizing them helps to clearly understand research trends, conceptual relationships, and emerging areas of interest in the field of gut microbiome and hypertension.

Table 7 lists the top 20 most frequently occurring keywords in the field of gut microbiome and hypertension. Besides “gut microbiome” (630 times), “hypertension” (312 times), and “blood pressure” (212 times), other frequently mentioned keywords in this study include “inflammatory response” (170 times), “short chain fatty acid” (154 times), “obesity” (140 times), “cardiovascular disease” (116 times), and “trimethylamine N-oxide.” This suggests that the hot research topics in the gut microbiome and hypertension are related to pathogenic mechanisms, gut microbiome metabolites, and clinical applications.

**Table 7:** Top 20 keywords in gut microbiome and hypertension.

Rank	Keyword	Occurrences	Total link strength	Rank	Keyword	Occurrence	Total link strength
1	Gut microbiome	630	4,378	11	Health	80	606
2	Hypertension	312	2,359	12	Diet	75	569
3	Blood pressure	212	1,692	13	Disease	73	525
4	Inflammatory response	170	1,401	14	Metabolism	73	561
5	Short chain fatty acid	154	1,348	15	Probiotics	71	594
6	Obesity	140	1,051	16	Mouse	66	442
7	Cardiovascular disease	116	1,012	17	Insulin resistance	64	519
8	Trimethylamine n-oxide	101	915	18	Oxidative stress	64	519
9	Dysbiosis	98	808	19	Heart failure	63	554
10	Risk factors	93	696	20	Gut microbial metabolites	59	539

**Figure 10:**
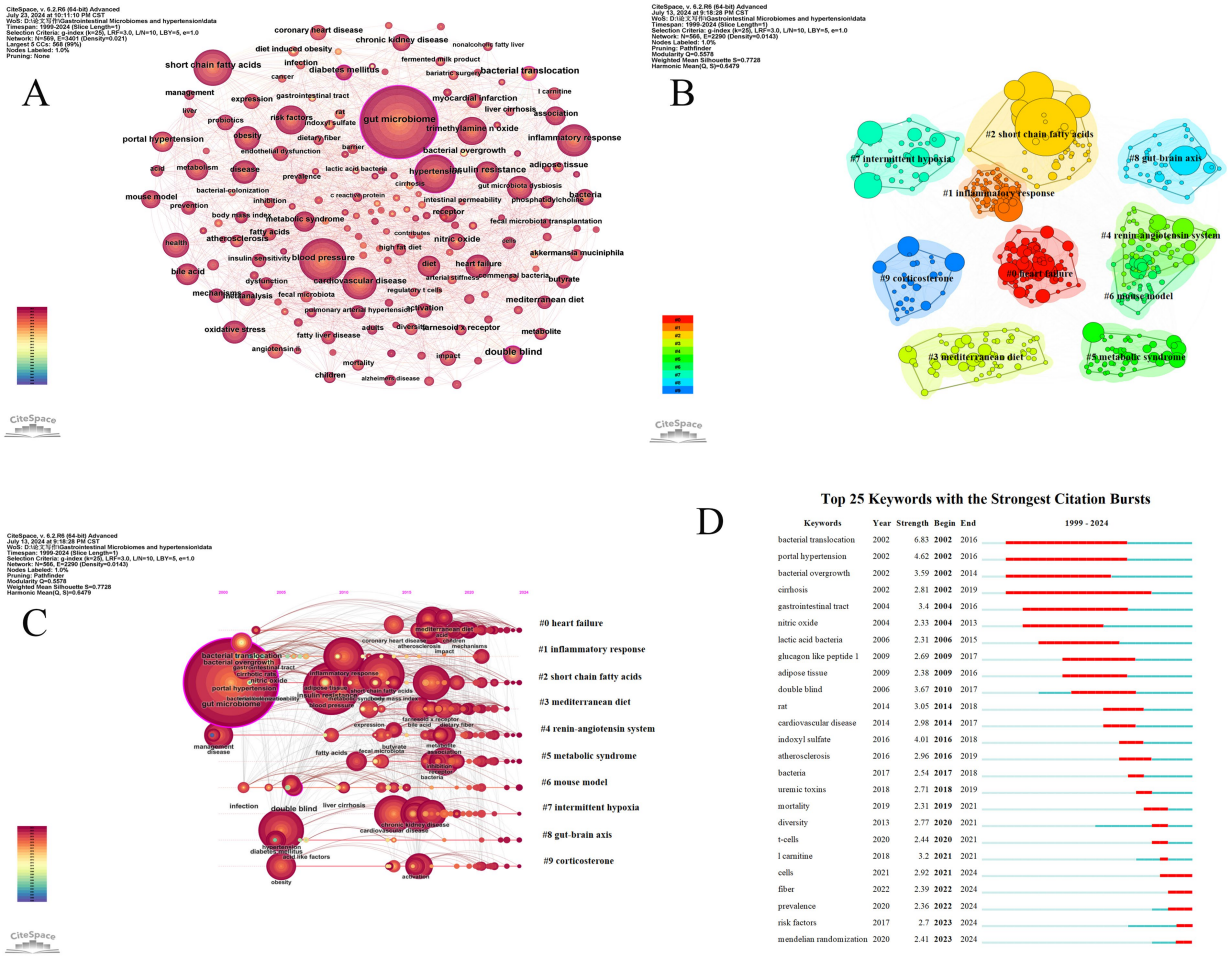
The analysis of keywords related to gut microbiome and hypertension. **(A)** The keyword co-occurrence map is generated by CiteSpace, where each node represents a keyword. The size of the node indicates the frequency of the keyword, and the thickness of the connections between nodes represents the frequency of co-occurrence of the keywords. **(B)** The keyword clustering map is generated by the LLR algorithm, resulting in 10 clustering labels. Each color cluster represents a different category, with the size of the cluster and the labels revealing the division of subfields. **(C)** The timeline chart related to gut microbiome and hypertension shows the temporal evolution of the 10 clusters. **(D)** The figure displays the top 25 keywords with the strongest burst, highlighting sudden shifts in information. The red bursts on the timeline represent these abrupt changes. These bursts indicate a rapid increase in keyword frequency and the emergence of important issues or solutions in the field.

Figure 10A shows the keyword map generated using CiteSpace. The parameter settings for CiteSpace are as follows: time slice (1999–2024), year per slice (1), term source (entire selection), node type (keyword), and selection criteria (top *N* = 50). All other parameters were set to their default values. Based on the co-occurrence keyword map, we created keyword clustering maps, a keyword timeline graph, and the top 25 keywords with the highest burst intensity.

Keyword clustering is based on the degree of association between keywords. In CiteSpace software, the Log-Likelihood Ratio (LLR) algorithm is used to generate keyword clustering maps. The more nodes in a cluster, the higher the research hotspot, and the smaller the cluster number. The modularity value (Q) and the average silhouette value (S) are two indicators used to evaluate the clustering effect in CiteSpace. When the Q value is greater than 0.3, it indicates that the detected community structure is significant; an S value greater than 0.5 suggests that the clustering is reasonable, and when the S value reaches 0.7, it indicates that the clustering efficiency is high and convincing (Yue et al., 2015). Figure 10B displays the keyword clustering map generated by CiteSpace, which includes 566 network nodes, 2,290 connections, a network density of 0.0143, an average silhouette value (S value) of 0.7728, and a clustering modularity value (Q value) of 0.5578. The top 10 clusters are presented in this study.

The timeline graph reveals the dynamic evolution of research hotspots represented by keywords, providing a clear view of research trends and emerging hotspots in the field. By conducting a log-likelihood ratio (LLR) test clustering analysis of co-occurring keywords, we created a timeline of keyword clusters (Figure 10C) that shows research trends over time in the gut microbiome and hypertension.

Keywords that suddenly emerge and are widely cited within a short period are called burst keywords. These are identified using the Kleinberg algorithm in CiteSpace. As an important indicator of frontier research hotspots, burst keywords can signal emerging trends. Figure 10D displays the top 25 keywords with the highest burst intensity. The results indicate that between 2002 and 2006, keywords such as “bacterial translocation,” “bacterial overgrowth,” “gastrointestinal tract,” “nitric oxide,” “lactic acid bacteria,” and “glucagon-like peptide 1” experienced significant bursts, suggesting that scholars during this period recognized a close relationship between gut microbiome migration, overgrowth, changes in the gastrointestinal microbiome, and hypertension. They also began using “lactic acid bacteria” and “glucagon-like peptide 1” to address dysbiosis. From 2017 to 2024, keywords such as “double blind,” “rat,” “cardiovascular disease,” “indoxyl sulfate,” “atherosclerosis,” “uremic toxins,” “l-carnitine,” and “Mendelian randomization” experienced high burst intensity, indicating promising development and highlighting that animal experiments have become a key method in research. Additionally, there is an increasing focus on the relationship between gut microbiome and cardiovascular diseases, diet, and the rising interest in Mendelian randomization as an emerging scientific research method.

## 4 Discussion

In this bibliometric analysis, we used CiteSpace, VOSviewer, and R-bibliometric to analyze 818 publications related to gut microbiome and hypertension. The data was retrieved from the Web of Science Core Collection database, covering publications from the start of the field up to July 10, 2024. The annual publication trend shows a gradual increase from 1999 to 2017, with a significant rise in the number of papers published annually since 2017, indicating growing research interest in the field. As gut microbiome research is in a critical development stage and the theoretical understanding of gut microbiome continues to mature, it is anticipated that the field will continue to advance in the coming years.

By analyzing the publication output from various countries/regions and institutions, we can identify those making significant contributions in the field. China and the United States are the top contributors, with 3 and 4 institutions, respectively, listed among the top 10 publishing organizations, as shown in Table 2. Major productive institutions from the US include the University of California system, University of Florida, and Vanderbilt University, while China’s top contributors are Capital Medical University, Central South University, Zhejiang University, and Peking Union Medical College. The US also leads in citation volume, with citations exceeding those from China by more than twice. The reasons for this may be attributed to the following factors: (a) The United States invests significantly in scientific research, with substantial funding and resources available, which may foster the production of high-quality research. (b) The United States is home to top-tier research institutions and universities, which contribute to the generation of innovative research and influential academic outcomes. (c) Research teams in the United States maintain close collaborations with numerous countries and regions, and the extensive exchange and cooperation are important factors in increasing citation counts. (d) The United States has maintained a leading position in various scientific fields for an extended period, accumulating a wealth of high-quality research outcomes. These results lay a solid academic foundation, making it easier for subsequent research to cite these foundational works. (e) Due to the database’s focus on publications from English-speaking countries, there may be a bias in the database’s coverage. Additionally, Germany and Spain are among the top 10 in publication volume and have high citation levels, indicating increasing global interest and high-quality research. The collaboration network shows that the US is the most connected country, underscoring its leading role in gut microbiome and hypertension. However, the overall trend is toward domestic collaboration, with international cooperation primarily occurring between developed countries. This suggests that scientific and research capabilities are closely linked to economic development. To advance the field, enhancing collaboration between countries and institutions, and sharing advanced technologies among leading organizations, is essential.

In the author’s analysis, Marques, Francine Z ranks first in total publications, citation volume, H-index, and link strength, indicating a highly influential role in the field and establishing her as a core researcher. Marques, Francine Z has focused on how dietary fiber intake affects gut microbiome and its metabolites to regulate blood pressure and provide cardiovascular protection (Marques et al., 2018; Marques et al., 2017), and she was the first to mechanistically confirm that dietary fiber can directly influence cardiovascular health (Marques et al., 2017). Following her are Li Jing from Capital Medical University and Kaye, David M from Monash University. Both have high citation volumes, H-indices, and link strength. Li et al. (2017) have demonstrated the causal relationship between abnormal gut microbiome and hypertension and emphasized the importance of early intervention for pre-hypertension patients. Kaye et al. (2020), who has a close collaborative relationship with Marques, Francine Z, has also conducted in-depth research on the relationship between dietary fiber, gut microbiome, and hypertension. The work of these authors has made significant contributions to advancing research on gut microbiome and hypertension.

As shown in Table 4, among the top 10 journals by publication volume, nine are in the JCR Q1 category. NUTRIENTS, INTERNATIONAL JOURNAL OF MOLECULAR SCIENCES, and HYPERTENSION are the top three journals with the highest number of publications, indicating their greater interest in research on gut microbiome and hypertension. All 10 journals with the highest citation counts are also in the JCR Q1 category, with Nature having the highest impact factor of 50.5. Notably, although the highest publication volume journals are primarily focused on gut microbiome and cardiovascular research, three of the top 10 co-cited journals (Nature, PNAS, Cell) are leading multidisciplinary journals. This suggests that these high-quality journals are key theoretical and data sources for researchers studying gut microbiome and hypertension and highlights the broad interdisciplinary and multi-disease scope of gut microbiome research.

### 4.1 Knowledge base of gut microbiome and hypertension

Co-citation refers to the situation where two papers are simultaneously cited by a subsequent paper. The two cited papers then form a co-citation relationship. The higher the co-citation frequency, the stronger the association between the two papers in terms of research topics, and it also indicates the significant influence these papers have in the research field. This analysis can reveal relationships between documents, reflect the knowledge structure and development trends of a research field, and help identify key literature, research hotspots, and academic networks. This, in turn, assists researchers in understanding significant findings and academic developments. Table 6 presents the top 10 highly cited documents, including eight experimental studies and two review articles, each exploring the relationship between gut microbiome and hypertension from different perspectives.

Li et al. (2017) published research in the journal Microbiome demonstrating that reduced bacterial diversity and changes in bacterial populations are closely associated with hypertension and prehypertension. Specifically, the proportion of Prevotella bacteria was found to be significantly higher in individuals with hypertension and prehypertension, suggesting that these bacteria may play an important role in the development of hypertension by triggering inflammatory responses. Additionally, Li Jing and colleagues observed that changes in the gut microbiome, including reduced bacterial amino acid biosynthesis, fatty acid utilization, and purine metabolism, as well as excessive production of LPS by gut bacteria, are directly related to the development of hypertension.

Marques et al. (2017) published a paper in Circulation that mechanistically explained the protective effects of dietary fiber on cardiovascular and kidney diseases. A high-fiber diet increased the production of short-chain fatty acid acetate, and dietary fiber and acetate regulation reduced the ratio between Firmicutes and Bacteroidetes, thereby improving gut dysbiosis. Fiber and acetate diets also inhibited cardiac and renal fibrosis, inflammation, and cardiac hypertrophy by downregulating the transcription factor Egr1. Additionally, the study demonstrated that fiber and acetate diets upregulated genes associated with circadian rhythms related to blood pressure, while downregulating RAS signaling in the kidneys and MAPK signaling in the heart.

Yang et al. (2015) research found that both hypertensive rats and patients exhibited significant reductions in gut microbial richness, diversity, and evenness. Notably, there was an increase in the Firmicutes/Bacteroidetes ratio and a decrease in bacteria producing acetate and butyrate. Using a hypertensive rat model, Yang verified that dimethylamino tetracycline can balance the hypertensive gut microbiome by lowering the Firmicutes/Bacteroidetes ratio, thereby exerting antihypertensive effects.

Wilck et al. (2017) found that high salt intake affects the gut microbiota in mice, particularly reducing the survival rate of *Lactobacillus murinus*, increasing T helper 17 cells (TH17), and consequently inducing elevated blood pressure. Kim S. et al. (2018) discovered that plasma levels of gut fatty acid-binding protein, lipopolysaccharides, and TH17 cells were significantly increased in hypertensive patients, indicating heightened gut inflammation and permeability. After butyrate treatment, the mucosal barrier in rats was strengthened, and changes in the microbiome influenced cardiac and vascular functions, suggesting a new approach for managing hypertension through manipulation of the gut microbiome and its barrier function.

Santisteban et al. (2017) discovered that in hypertensive rat models, there is enhanced sympathetic neuronal communication between the hypothalamic paraventricular nucleus (PVN) and the gut. This dysfunctional sympathetic-gut communication is closely associated with increased intestinal epithelial barrier permeability, inflammatory immune responses, and microbiome dysregulation, playing a key role in the progression of hypertension. Bartolomaeus et al. (2019) found that the gut microbiome metabolite propionate, mediated by regulatory T cells (Tregs), reduced the frequency of splenic effector memory T cells and splenic TH17 cells, as well as cardiac immune cell infiltration in hypertensive mouse models, demonstrating anti-inflammatory, anti-atherosclerotic, and anti-hypertensive effects on cardiac remodeling. Marques et al. (2018) published a review in Nature Reviews Cardiology on the various mechanisms by which gut microbiome dysregulation induces hypertension. Notably, Marques proposed the potential role of antibiotics in treating resistant hypertension and suggested using the gut microbiome and its metabolites as early biomarkers for hypertension and cardiovascular diseases. Marques’s comprehensive analysis and scientific hypotheses on the gut microbiome and hypertension have made significant contributions to the field.

Adnan et al. (2017) have experimentally demonstrated a clear causal relationship between gut microbiome dysregulation and hypertension. In hypertensive rats, the ratio of Firmicutes to Bacteroidetes in the gut microbiome is significantly elevated, which indicates dysregulation of the gut microbiome. W.H. Wilson Tang and colleagues summarized various mechanisms through which the gut microbiome affects the pathogenesis of cardiovascular diseases in the host. These include metabolite-dependent pathways such as the trimethylamine/trimethylamine N-oxide pathway, short-chain fatty acid pathways, and primary and secondary bile acid pathways, as well as non-metabolite-dependent pathways such as bacterial translocation and the entry of bacterial products into the systemic circulation, which induce inflammatory responses (Tang et al., 2017).

The top 10 most-cited papers have revealed and summarized significant knowledge in the field of gut microbiome and hypertension. Eight of these papers explore the pathological mechanisms by which gut microbiome dysregulation promotes the development and progression of hypertension. Four of these studies discuss the future therapeutic potential of targeting gut microbiome and its related mechanisms to improve hypertension, while two papers focus on the bidirectional effects of diet on gut microbiome and hypertension. These 10 papers cover the full spectrum from proving the causal relationship between gut microbiome and hypertension, to understanding the mechanisms through which gut microbiome affects hypertension, and to exploring the potential of targeting gut microbiome in hypertension treatment. They reflect a high level of academic achievement in the field and serve as an important knowledge base, providing essential insights and research directions for future studies.

### 4.2 Identified research hotspots and emerging topics

Keywords represent the core content of publications, and the evolution of keywords reflects the changing trends in research hotspots within a field. Keyword clustering encompasses research content related to specific topics. Keyword co-occurrence refers to the frequency with which two or more keywords appear together in the same paper. Essentially, it reflects the semantic association of knowledge units through keyword co-occurrence. High-frequency keywords indicate ongoing, highly focused topics within the field. In hotspot and frontier analysis, Figure 8 visually shows the emergence, changes, and development of keywords in the field of gut microbiome and hypertension research. Combined with the top 20 high-frequency keywords shown in Table 7 and the top ten keyword clusters shown in Table 8, this helps identify the developmental trajectory and future research trends in the field.

**Table 8:** Ten keyword clusters.

Cluster groups	Keywords
#0 heart failure	Heart failure, coronary heart disease, myocardial infarction, atherosclerosis, pulmonary arterial hypertension, dysfunction, arterial stiffness, cardiovascular risk, cardiometabolic risk, cardiovascular health, myocardial fibrosis
#1 inflammatory response	Inflammatory response, nitric oxide, innate immunity, necrosis factor alpha, lipopolysaccharide, tumor necrosis factor, host defense, cytokine-generating organ, kappa-b
#2 short chain fatty acids	Short chain fatty acids, gut microbiome, probiotics, trimethylamine n oxide, bile acid, bacterial-colonization, glucagon like peptide 1, escherichia-coli, butyrate producing bacteria
#3 mediterranean diet	Mediterranean diet, dietary patterns, dietary fiber, dietary interventions, dietary fiber intake, bifidobacterium-bifidum
#4 renin-angiotensin system	Renin-angiotensin system, endothelin receptor antagonist, insulin resistance, endothelial dysfunction, angiotensin-converting enzyme 2 (ace2), angiotensin ii,
#5 metabolic syndrome	Metabolic syndrome, adult metabolic diseases, fatty liver disease, diabetes mellitus, alcoholic liver disease, metabolic disorders
#6 mouse model	Mouse model, meta analysis, mendelian randomization, randomized controlled trial, epidemiology, clinical research studies, double blind, metabolome, mass-spectrometric analyses
#7 intermittent hypoxia	Intermittent hypoxia, obstructive sleep apnea, asymmetric dimethylarginine, oxidative stress, endothelial function, antioxidant, chronic intermittent hypoxia
#8 gut-brain axis	Gut-brain axis, intestinal barrier, protein coupled receptor, nervous system, epithelial barrier, epithelial tight junctions, microbiota-gut-brain axis
#9 corticosterone	Carboxykinase gtp gene, 11 beta-hsd2, 11 beta-hsd1, cortisol, asymmetric dimethylarginine

#### 4.2.1 The relationship between gut microbiome and hypertension

Past research has made significant progress in understanding the relationship between gut microbiome and hypertension. Both hypertension and prehypertension patients show substantial changes in gut microbiome compared to healthy individuals. Li et al. (2017) used metagenomic and metabolomic analyses to study the gut microbiome structures in healthy people, prehypertensive individuals, and hypertensive patients. They found that both prehypertensive and hypertensive patients had significantly reduced gut microbiome abundance, diversity, and gene count compared to healthy individuals. Further experiments revealed that germ-free mice receiving FMT from hypertensive human donors exhibited increased blood pressure (Li et al., 2017). A cohort study on the relationship between gut microbiome and hypertension found that hypertensive patients had a higher proportion of Firmicutes compared to healthy individuals (Sun et al., 2019). Hypertension has also been shown to alter the microbiota of the cecum and its contents (Evans et al., 2023). A cross-sectional study in Hong Kong found that gut microbiome dysbiosis in women was associated with 24-h dynamic blood pressure fluctuations. Hypertensive women had significantly higher levels of Gnavus rumen bacteria, Bolteae spore-forming bacteria, and *Bacteroides ovatus*, while normotensive women had higher levels of *Dorea formicigenerans*. Interestingly, no significant correlation was found between bacterial species changes and blood pressure fluctuations in men (Virwani et al., 2023). Similarly, animal models have shown that SHR have nearly five times the ratio of Firmicutes to Bacteroidetes compared to WKY rats (Yang et al., 2015; Adnan et al., 2017). In two hypertension mouse models induced by angiotensin II and deoxycorticosterone acetate, a significant decrease in gut microbiome richness was also observed (Yang et al., 2015; Adnan et al., 2017). A study indicates that fermented Lactobacillus is a key biomarker for distinguishing severe preeclampsia from healthy controls. Subsequent untargeted fecal metabolomics analysis found that higher concentrations of phenylpropionic acid and guanidinoacetic acid were associated with an increased abundance of fermented Lactobacillus, suggesting that changes in the gut microbiota may lead to metabolite alterations, potentially triggering severe preeclampsia (Liu et al., 2024). Mendelian randomization, an innovative statistical method, offers a significant advantage in inferring potential causal relationships between exposure factors and outcomes. Multiple Mendelian randomization studies have found a definite causal relationship between gut microbiome and its metabolites with blood pressure (Li et al., 2023; Zhao et al., 2023; He et al., 2023). These findings indicate a close relationship between gut microbiome and hypertension, suggesting a bidirectional causal relationship. However, human research is still limited by confounding factors (such as diet, medication, etc.) and insufficient longitudinal evidence. Research into the relationship between gut microbiome and hypertension is the beginning of the field’s development and paves the way for subsequent studies on how gut microbiome participates in blood pressure regulation.

#### 4.2.2 Mechanisms of gut microbiome involvement in blood pressure regulation

There is growing evidence supporting the role of gut microbiome in the development and progression of hypertension, and research on its pathological mechanisms has made significant strides since the early days of the field. This study used keyword co-occurrence and clustering analysis to identify key mechanisms of gut microbiome dysbiosis, including inflammation and immune response induced by gut microbiome and its metabolites, gut barrier damage, and excessive sympathetic nerve activation.

A wealth of research indicates that inflammatory immune responses are a major cause of hypertension (Drummond et al., 2019), with the gut being the largest immune organ and containing the highest number of immune cells in the body (Kuziel and Rakoff-Nahoum, 2022). The gut plays a crucial role in various immune pathways (Kuziel and Rakoff-Nahoum, 2022). Gut microbiome function is considered a potential mediator of gut immune function, involving immune cell activation, release of inflammatory factors, and the formation of an inflammatory microenvironment (Riazi-Rad et al., 2021). Notably, several immune cell types that either promote or regulate hypertension are directly influenced by changes in the microbiota or microbiota-dependent signaling pathways. Additionally, the gut microbiome participates in the formation of the gut mucosal barrier and interacts with mucosal immune cells to modulate the immune system (Kim, 2021; Selvakumar et al., 2022). Current research indicates that gut microbiome mainly regulates inflammation through two mechanisms: first, by being recognized by immune cells, which induces either innate or adaptive immune responses (Lavelle et al., 2010; Steimle and Frick, 2016). Dysbiosis and accompanying gut barrier damage can be recognized by innate immune cells, leading to excessive activation of these cells and the release of large amounts of inflammatory factors, resulting in systemic inflammation (Li et al., 2022; Wang et al., 2023). Second, gut microbiome metabolites, such as short-chain fatty acids, indoleacetic acid, bile acids, and trimethylamine N-oxide (TMAO), can alter the phenotype and function of immune cells, either inhibiting or activating inflammation-related pathways to modify the inflammatory state (Wang et al., 2022; Mao et al., 2022; Ilyas et al., 2022; Su et al., 2022).

Short-chain fatty acids (SCFAs) are important gut microbiome metabolites that play crucial roles in regulating immune responses, enhancing intestinal epithelial barrier function, and maintaining gut homeostasis (Wang et al., 2023; Wu et al., 2017). Specific researches on SCFAs have shown that butyrate induces the production of interleukin-10 (IL-10) and regulatory T cell (Treg) differentiation through the activation of GPR109a, thereby suppressing inflammatory responses. Additionally, butyrate reduces IL-1β, IL-6, and tumor necrosis factor-alpha (TNF-α) through the TLR4 pathway, collectively contributing to the attenuation of host inflammatory responses (Feingold et al., 2014; Sam et al., 2021).

Lipopolysaccharides (LPS), which are glycolipids in the outer membrane of Gram-negative bacteria, can increase due to gut microbiome dysbiosis. These LPS can enter the bloodstream through an enhanced permeable intestinal epithelial barrier (Yang et al., 2023). LPS is captured by lipopolysaccharide-binding protein (LBP) and transported to membrane receptor CD14 on immune cell surfaces. The LPS-CD14 complex then binds to the TLR4 protein and the lymphocyte antigen (MD-2) complex, activating intracellular signaling pathways involving Myeloid Differentiation factor 88 (MyD88) and interleukin receptor-associated kinase (IRAK), leading to the release of numerous inflammatory factors and contributing to systemic inflammation and accelerated hypertension (Di Lorenzo et al., 2022; Hartmann et al., 2020).

Trimethylamine-N-oxide (TMAO) is a gut microbiome -dependent metabolite of L-carnitine, choline, and phosphatidylcholine. TMAO is considered a switch for activating pro-inflammatory cascades. High levels of TMAO can increase the expression of inflammatory chemokines, adhesion molecules, and cytokines, thereby participating in the development of vascular inflammation and endothelial dysfunction, which affects hypertension (Chen et al., 2019; Witkowski et al., 2022).

Indole-3-acetic acid (IAA), a metabolite of tryptophan, can be directly converted by gut microbes. These metabolites can activate signaling pathways through transcription factors such as the aryl hydrocarbon receptor (AhR), controlling immune cell differentiation and function (Su et al., 2022), thus maintaining immune cell environment balance and function (Hill and Artis, 2010). Studies have found that microbially-derived indole derivatives have a protective role in blood pressure regulation (Paeslack et al., 2022).

Bile acids are important components of bile and are converted from primary bile acids to secondary bile acids by gut microbes (Mistry et al., 2017). Bile acids can directly act on dendritic cells, regulate peripheral Treg cell differentiation, and activate inflammasomes, thereby exerting anti-inflammatory effects (Campbell et al., 2020). Additionally, bile acids can function as signaling molecules, activating G protein-coupled receptor (TGR5) to influence cAMP signaling-mediated NF-κB activation, inhibiting the expression and secretion of pro-inflammatory factors (Yoneno et al., 2013). Beyond their role in immune and inflammatory responses, bile acids also act as endogenous vasodilators, directly promoting NO production and inhibiting endothelin-1 (ET-1) release to exert antihypertensive effects (Fiorucci et al., 2017). A deeper understanding of the intestinal inflammatory immune response allows us to develop treatment strategies in a more comprehensive and objective manner. We can foresee that targeting the inflammatory immune response, inhibiting excessive inflammation, and reconstructing immune homeostasis have significant potential in the treatment of hypertension. It is noteworthy that supplementing with SCFA-producing strains or directly adding SCFAs to stimulate bile acid secretion to activate Treg cells, or selecting inhibitors or probiotics based on the patient’s microbiome characteristics, may also play an important role in the future treatment of hypertension.

Blood pressure in humans is regulated by both the sympathetic and parasympathetic nervous systems. The vagus nerve is a key regulatory communication pathway between the gut microbiome, the gut, and the central nervous system. Microbiota-derived signals can reach the central nervous system either directly or indirectly (Walsh and Zemper, 2019). The central nervous system responds to these signals by modulating the activity of the sympathetic and vagal branches of the autonomic nervous system (ANS) as well as the hypothalamic–pituitary–adrenal (HPA) axis (Martin et al., 2018; Yano et al., 2015). Research has shown that short-chain fatty acids (SCFAs) can bind to G protein-coupled receptors (GPCRs), such as GPR41, GPR43, GPR109A, and olfactory receptor 78 (Olfr8), to initiate signaling (Mann et al., 2024). These receptors are also present in sympathetic ganglia. SCFAs can directly regulate the sympathetic nervous system by acting on receptors expressed in the sympathetic ganglia and influence gut neurofeedback through receptors expressed in the vagus nerve (Nøhr et al., 2015; Zubcevic et al., 2019). This suggests that communication between the gut and brain is bidirectional and plays a role in blood pressure regulation. The research by Onyszkiewicz supports this view (Onyszkiewicz et al., 2019). They found that hypertension in rats treated with butyrate was reduced, but the antihypertensive effect was diminished when the subdiaphragmatic vagus nerve was cut (Onyszkiewicz et al., 2019). Similarly, other researchers observed that transferring the fecal microbiota from SHR rats to WKY rats resulted in increased blood pressure in the WKY rats, along with enhanced systemic sympathetic activity, neuroinflammation, and the presence of reactive oxygen species in the paraventricular nucleus (Toral et al., 2019). Intestinal microbiota and its metabolites regulate blood pressure through a neuro-metabolic-immune multidimensional network, with mechanisms that exhibit dynamic interactions. In the future, it will be necessary to use systems biology approaches to analyze the temporal and spatial specificity of the microbiome-host interaction, while also promoting interdisciplinary collaboration to achieve a closed-loop translation from mechanisms to interventions.

The gut barrier function consists of a complex, multi-layered system, including various types of intestinal cells, junctions, and proteins (Ghosh et al., 2020). This barrier is based on tight junctions between cells and plays a role in the symbiotic relationship between the host and microorganisms. The gut microbiome also helps regulate gut barrier function through the production of metabolites like SCFAs, which contribute to maintaining gut homeostasis and preventing pathogen spread. When the gut barrier function is compromised, intestinal epithelial cells are exposed to gut microbes and their metabolites, which can trigger immune activation and inflammatory responses (Bischoff et al., 2014). Additionally, bacteria and bacterial products can enter systemic circulation through dysfunctional paracellular transport pathways, leading to systemic and gut diseases, including hypertension (Chelakkot et al., 2018). Researches show that hypertensive patients have significantly higher levels of LPS, zonulin, plasma intestinal fatty acid-binding protein (I-FABP), and diamine oxidase in their blood compared to those with normal blood pressure (Kim S. et al., 2018; Li et al., 2020; Ntlahla et al., 2021). Moreover, gut barrier function declines exponentially with the progression of chronic hypertension (Vemuri et al., 2022), suggesting that impaired gut barrier function could serve as a marker for monitoring hypertension. While research on the gut-brain-microbiome axis and its relation to hypertension is increasing, limitations in detection methods and marker inference still leave many questions unanswered. However, with advancements in marker detection and gene sequencing, the specific effects of the gut-brain-microbiome axis on hypertension, differences in how various microbiota communities regulate gut barrier function, and clinical translation hold significant research potential and may become future focal points.

#### 4.2.3 Targeting gut microbiome for hypertension interventions

Based on keyword co-occurrence maps and clustering results, targeting gut microbiome to intervene in hypertension has become a research hotspot, and we predict this will be a key focus of future studies in this field.

Fecal microbiota transplantation (FMT) involves transferring functional bacteria from the feces of a donor (either healthy or diseased) into the gastrointestinal tract of a recipient to reconstruct the recipient’s gut microbiome (Yang et al., 2023). Initially, this method was used to investigate the relationship between gut microbiome and hypertension, but recent years have seen growing interest in using FMT for hypertension treatment. Studies show that FMT can achieve an 80% effectiveness rate in treating drug-resistant *Clostridium difficile* infections (Colman and Rubin, 2014). Additionally, transferring fecal bacteria from healthy mice to Ang II-induced hypertensive mice can also reduce the systolic blood pressure in the hypertensive mice (Kim T. T. et al., 2018). However, the use of FMT for treating hypertension is limited by the high requirements for the host. Compared to fecal material transplantation, transferring a specific bacterial group may be a better choice for improving disease states (Wymore Brand et al., 2015).

Although the use of antibiotics for hypertension is somewhat controversial, there is a growing body of research supporting its feasibility. A study on resistant hypertension patients showed that minocycline reduced daytime systolic blood pressure by decreasing gut inflammatory cell populations (Pepine et al., 2021). Similarly, amoxicillin was found to lower blood pressure in SHR rats and alter gut microbiome, with this effect persisting even after discontinuation of the antibiotic treatment (Galla et al., 2020). Research also indicates that pre-treating hypertensive rats with antibiotics to reduce gut microbiome enhances their response to the angiotensin-converting enzyme inhibitor captopril (Kyoung and Yang, 2022). Robles-Vera et al. (2021) found that doxycycline decreased systolic blood pressure in DOCA-salt rats, improved endothelial dysfunction, reduced aortic oxidative stress and inflammation, and restored Treg levels in the aorta. While antibiotic treatment for hypertension has been validated in animal studies, clinical evidence remains relatively limited. Considering the potential risks of long-term antibiotic use, further research is needed to assess the feasibility of this approach.

Diet is the most significant modifiable factor in controlling obesity and related diseases. Dietary fiber has been shown by multiple studies to regulate gut microbiome and blood pressure (Petrick et al., 2023; Xu and Marques, 2022), while high-salt and high-fat diets have the opposite effect (de Araújo Henriques Ferreira et al., 2022; Canale et al., 2021). Vitamin C has also been found to regulate gut microbiome and gut-brain axis dysregulation by reducing inflammation and oxidative stress (Li Y. et al., 2021). Consuming table olives not only lowers plasma levels of malondialdehyde and angiotensin II in SHR, but also remodels gut microbiome associated with antihypertensive activity by decreasing Peptoniphilus and increasing Akkermansia, Sutterella, Allobaculum, Ruminococcus, and Oscillospira. This also enhances the growth of beneficial probiotics such as Lactobacillus and Bifidobacterium (Gómez-Contreras et al., 2023). Exercise can improve hypertension, and a recent study found that in exercise-trained SHR, the number of activated microglia decreased, neuroinflammation in the hypothalamic paraventricular nucleus, gut pathology, inflammation, and gut barrier function all improved, suggesting that exercise lowers blood pressure by influencing the gut-brain axis (Xia et al., 2021). Natural medicines are also receiving increasing attention for their effects on improving gut microbiome. An animal study found that *Garcinia dulcis* extract affected the gut microbiota and metabolite profile in hypertensive rats, indicating its antihypertensive properties (Sornchuer et al., 2023). Evans et al. found that rhein impacted the microbiota in the feces and colon of hypertensive rats, and data suggested that rhein alleviated pathological cardiac hypertrophy in female hypertensive rats, though the relationship between these effects requires further research (Evans et al., 2023). Li B. et al. (2021) found that micro-powder of Dendrobium officinale improved gut microbiome, increased the production, transport, and utilization of short-chain fatty acids (SCFAs), activated the SCFA-GPCR43/41 axis in the gut and vasculature, improved endothelial function, and reduced blood pressure in metabolic hypertensive rats. The impact of diet, natural medicines, and lifestyle on the gut microbiota and hypertension is receiving increasing attention from scholars. However, current research remains just the tip of the iceberg in this field. The complex relationships between host genetics, dietary and lifestyle habits, and the gut microbiome still require larger-scale basic and clinical studies. Further research should focus on how different dietary lifestyles or natural medicines affect the abundance of gut microbiota in hypertensive patients, in order to achieve personalized treatment strategies.

In the research on gut microbiome and hypertension, scholars have shifted their focus from merely exploring the association between gut microbiome and hypertension to understanding the causal relationship. With advancements in techniques such as microbiota characterization, quantitative analysis, and gene sequencing, researchers have delved deeper into the pathological mechanisms through which gut microbiome induces hypertension. They have discovered that gut microbiome contributes to the development of hypertension through various mechanisms, including gut microbiome dysbiosis, gut barrier damage, increased neuro-sympathetic activity, and gut microbiome metabolic products. As the pathological mechanisms become more evident and well-defined, the application value of targeting gut microbiome for hypertension treatment has gained increasing attention and is emerging as a popular research direction. Randomized controlled trials (RCT) are considered the gold standard for providing the highest level of etiological evidence in medical research. However, RCTs have notable drawbacks, including high costs, ethical constraints, and significant limitations in evaluating long-term health effects, as well as the difficulty of controlling for confounding factors such as diet and lifestyle. Mendelian randomization (MR) is a research design used to infer causal relationships, utilizing genetic variation as an instrumental variable (IV) to assess the impact of exposure on outcomes. With the discovery of numerous genetic variations closely related to specific traits in the biological field, coupled with the release of large-scale genome-wide association study (GWAS) data sets containing hundreds of thousands of summaries on the relationship between exposures, diseases, and genetic variations, MR has gained widespread application, particularly in the study of causal relationships between hypertension and the gut microbiome and its related factors. This has brought new momentum to research in this area. We predict that more in-depth and large-scale MR studies will guide more precise intervention strategies, and the integration of MR with multi-omics approaches, such as metabolomics, proteomics, and epigenetics, will enable more accurate predictions of the complete causal network of microbiome-host interactions.

#### 4.2.4 Issues and future prospects of gut microbiome in the field of hypertension

Despite significant progress in understanding the relationship between gut microbiome and hypertension, our research reveals several unresolved issues in this field: (a) Although numerous studies indicate that the relationship between gut microbiome and hypertension often involves inflammatory immune responses, our understanding of gut microbiome is still just scratching the surface. The 16S rRNA sequencing method is widely used for gut microbiome sequencing, but to further explore its mechanisms, more advanced research methods are needed. While whole-genome sequencing has been used in experiments, its high cost has prevented it from becoming widely popular. (b) Although the relationship between hypertension and gut microbiome is established, there is still a need to identify and characterize specific bacterial strains related to hypertension. (c) Current public human microbiome datasets lack diversity; most existing public data come from European and American countries (Abdill et al., 2022), while hypertension is more prevalent in low- and middle-income countries. The association between microbiome characteristics and hypertension may be affected by the lack of diverse sample data. (d) Gut microbiome is influenced by genetic and sex factors. The mechanisms through which genetic factors affect gut microbiome and the potential impacts of different sexes on the gut microbiota of hypertension patients require further investigation. (e) The potential of gut microbiome as a dynamic biomarker has not been fully developed. Its time-dependent fluctuations (such as circadian rhythms, antibiotic disturbances, etc.) and their long-term association with blood pressure regulation still require further exploration. (f) Targeting gut microbiome for hypertension treatment has become an emerging hot topic in the field, but research primarily focuses on animal models of hypertension, with a lack of related clinical randomized controlled trials. However, with continuous advancements in scientific research and decreasing sequencing costs, further discoveries and insights are expected.

Although the role of the gut microbiome in the pathophysiology of hypertension is gradually becoming clearer, its clinical translation remains limited by the scarcity and heterogeneity of interventional trials. This translational gap may also make it more difficult for mechanistic research to serve clinical trials, as factors such as differences between animal models and human physiology, the dynamic nature of microbiome-host interactions, and the influence of confounding factors cannot be fully validated. To accelerate the translation from mechanisms to clinical applications, this study recommends the following priority strategies: (a) Expanding sample diversity and long-term follow-up, establishing a standardized microbiome intervention platform through international collaborations, and including populations from different racial and dietary backgrounds. (b) Integrating multi-omics approaches such as metagenomics, metabolomics, and immunomics with real-world data in clinical trial research. (c) Promoting interdisciplinary translational medicine collaboration, such as linking artificial intelligence in biomedical technology with medical practice. At the same time, it is undeniable that, based on the findings of this study, the majority of the literature is concentrated in high-income countries. While there is a large population of hypertension patients in low- and middle-income countries, the lack of high-impact publications in these regions can be attributed to multiple factors, such as limited research funding and academic environments. This has led to a significant geographical and economic imbalance in research within this field. Such bias may restrict the generalizability and translational value of the research due to the unique microbiome characteristics shaped by regional dietary patterns, environmental exposures, and genetic backgrounds, as well as the development of corresponding intervention strategies. Future research should aim to overcome geographical limitations by seeking international collaborations, establishing an inclusive global microbiome alliance, incorporating representative population samples from different regions, and promoting the development of data-sharing platforms.

This study, through bibliometric analysis, reveals the research trends in the field of gut microbiota and hypertension. Its core findings not only deepen the understanding of the pathological mechanisms but also highlight the crucial role of interdisciplinary collaboration in advancing precision medicine. The high-frequency keywords and emerging terms identified in this study, such as “Mendelian randomization,” indicate that research on microbiomes and hypertension has entered a data-driven phase. The heterogeneity of the identified keyword terms also facilitates the development of unified data annotation standards through collaboration between experts in the cardiovascular and microbiome fields. Furthermore, based on the results of this study, scholars in the field of data science, along with experts in cardiovascular and microbiome research, can deepen their cooperation, incorporate more data models, and further expand this bibliometric framework to conduct comparative analyses of gut microbiota characteristics in hypertension-related comorbidities such as metabolic syndrome, chronic kidney disease, and preeclampsia. A cross-disease bibliometric map could uncover shared pathophysiological foundations or distinct microbiome-disease networks, ultimately guiding the development of multi-target therapies to address the systemic interactions between hypertension and its related diseases.

## 5 Limitation

It should be acknowledged that this study still has certain limitations. First, due to the adaptability of the software, this study only included literature from the Web of Science Core Collection database and did not fully cover literature from Scopus, other regional databases, and open-access platforms, which limited the coverage of novel findings or research from non-English-speaking countries. Secondly, the search strategy was limited to English-language literature, which may have overlooked important regional research findings published in other languages, particularly non-English studies from high-incidence hypertension regions. This introduces a risk of language bias, which could result in regional knowledge gaps, a lack of diversity in mechanisms, and the tendency of non-English-speaking scholars to publish in local journals due to language barriers. As a result, their contributions may remain “invisible” within the international collaboration network. Future research will incorporate multi-source data, multi-language searches, and mixed methods (e.g., including expert interviews) to enhance the completeness and richness of the knowledge graph in this field.

## 6 Conclusion

This study used CiteSpace, VOSviewer, and the R package “bibliometric” to conduct a comprehensive analysis of the distribution of countries/regions, institutions, and authors in the field of gut microbiome and hypertension from 1999 to 2024. It also analyzed and predicted high co-cited literature, research hotspots, and development trends. Our research provides a thorough overview of the basic concepts and current status in gut microbiome and hypertension research, and delves into frontier areas and future trends. These findings will enhance understanding of the field and guide future research efforts.

## Data Availability

The original contributions presented in the study are included in the article/Supplementary material, further inquiries can be directed to the corresponding authors.
